# Investigating the delivery of health and nutrition interventions for women and children in conflict settings: a collection of case studies from the BRANCH Consortium

**DOI:** 10.1186/s13031-020-00276-y

**Published:** 2020-05-27

**Authors:** Anushka Ataullahjan, Michelle F. Gaffey, Samira Sami, Neha S. Singh, Hannah Tappis, Robert E. Black, Karl Blanchet, Ties Boerma, Ana Langer, Paul B. Spiegel, Ronald J. Waldman, Paul H. Wise, Zulfiqar A. Bhutta

**Affiliations:** 1 Centre for Global Child Health, Hospital for Sick Children, Toronto, Canada; 2 Center for Humanitarian Health, Johns Hopkins Bloomberg School of Public Health, Baltimore, MD USA; 3 Health in Humanitarian Crises Centre, London School of Hygiene and Tropical Medicine, London, UK; 4 Johns Hopkins Bloomberg School of Public Health, Baltimore, MD USA; 5 University of Manitoba, Winnipeg, Canada; 6 Women and Health Initiative, Department of Global Health and Population, Harvard TH Chan School of Public Health, Boston, USA; 7 Milken Institute School of Public Health, George Washington University, Washington DC, USA; 8 The Center for Policy, Outcomes and Prevention, Stanford University, Palo Alto, CA USA; 9 Center of Excellence in Women and Child Health, Aga Khan University, Karachi, Pakistan

**Keywords:** Conflict, Humanitarian, Women, Newborn, Child, Adolescent, Health

## Abstract

Globally, the number of people affected by conflict is the highest in history, and continues to steadily increase. There is currently a pressing need to better understand how to deliver critical health interventions to women and children affected by conflict. The compendium of articles presented in this *Conflict and Health* Collection brings together a range of case studies recently undertaken by the BRANCH Consortium (Bridging Research & Action in Conflict Settings for the Health of Women and Children). These case studies describe how humanitarian actors navigate and negotiate the multiple obstacles and forces that challenge the delivery of health and nutrition interventions for women, children and adolescents in conflict-affected settings, and to ultimately provide some insight into how service delivery can be improved.

## Main text

Over the last decade, the number of people affected and displaced by armed conflict has reached unprecedented highs [1]. Armed conflict has severe implications for health through the increased risk of injury and death, and a multitude of indirect effects [2, 3]. Armed conflict also has significant negative effects on public health systems, limiting access to and the quality of preventive and curative services [4]. Moreover, disregard for the humanitarian principles of humanity, neutrality, impartiality, and independence by a range of armed actors due to the changing nature of warfare has contributed to attacks against health workers and facilities, further compromising the delivery of services [5]. Aerial bombardment and artillery fire often damages health infrastructure such as hospitals and health centres directly, as well as affecting water and electricity infrastructure that then threatens the operation of health facilities [6, 7]. The longstanding impacts of conflict continue even after the violence has subsided [8], including displacement, which can expose individuals to increased risk of violence, malnutrition, and infectious disease [9]. Although conflict has health impacts on populations at large, evidence demonstrates that the health effects of conflict on women and children are often neglected [10–13], necessitating a more focused approach to address the health concerns of conflict-affected women and children.

Humanitarian actors face numerous barriers when attempting to deliver critical health services to conflict-affected populations. Violent attacks against aid workers has become a common risk during humanitarian responses in conflict settings [14–16]. Emerging needs in conflict settings such as the Ebola epidemic in DRC and cholera outbreak in Yemen diverted attention and funding at the expense of continuing essential sexual, reproductive, maternal, newborn, child, and adolescent health and nutrition (SRMNCAH & N) services. Although global standards and guidelines for clinical practice or public health policy have been implemented in stable contexts, adapting these to conflict-affected settings can prove challenging. The large variability in local sociopolitical contexts, degrees of health system capacity across rapidly changing conflict situations, and different population movement and security scenarios all complicate the development and implementation of standard guidelines for such settings. Increasingly, displaced populations live outside of camps and in urban settings, which are not the settings for which most existing protocols for emergency operations were developed [17]. The high risk of slipping back into conflict, with some estimating a 40% risk of a post-conflict country returning to conflict within a decade after deaths fall below a specific threshold, further reflects ways in which the nature of conflict has changed [18]. Guidance that was primarily designed for ‘acute’ conflicts needs to be revisited to address these changes, in particular, the increasing number of ‘protracted’ conflicts and the extended periods of time that individuals remain displaced [19].

Despite growing academic interest in the humanitarian space, the need for further research persists [20]. More specifically, there is a need for research that describes and evaluates strategies to overcome delivery barriers and adapt services to the specific resource and security constraints of modern armed conflict, and its extended implications for affected populations and health systems [20–22]. The compendium of articles presented in this *Conflict and Health* Collection brings together a range of case studies recently undertaken by members and partners of the BRANCH Consortium (Bridging Research & Action in Conflict Settings for the Health of Women and Children) [23]. These case studies aim to better understand how humanitarian actors navigate and negotiate the multiple obstacles and forces that challenge the delivery of health and nutrition interventions for women, children and adolescents in conflict-affected settings, and to ultimately provide some insight into how service delivery can be improved.

Case studies were conducted in Afghanistan, Colombia, Democratic Republic of Congo (DRC), Mali, Nigeria, Pakistan, Somalia, South Sudan, Syria, and Yemen. These study settings were selected by the BRANCH co-investigators following a technical workshop held at the Harvard T.H. Chan School of Publice Health in 2017 to agree on the selection criteria and a shortlist of types of conflict situations. These 10 countries were ultimately selected to ensure variability in geographic region, conflict phase, type and extent of population displacement, national income, and existing level of documentation and attention to SRMNCAH&N in these settings in the literature. Additionally, the study of the delivery of health and nutrition interventions for women and children in each of these 10 settings was deemed to be feasible in terms of likely data availability and the existence of experienced and interested local research partners. Once countries were selected, the BRANCH co-investigators formalized local research partnerships, with local co-investigators then playing a key role in selection of specific case study sites and developing country specific protocols.

All case study teams applied a mixed-methods approach that included literature and document review, secondary analysis of existing quantitative data, and primary qualitative data collection and analysis based on a common protocol developed by the BRANCH Consortium investigators (see Additional file 1). The common protocol outlined the objectives and research questions that all case studies would attempt to address and included a standardized set of intervention coverage indicators for which data and information would ideally be sought and analysed, as well as a set of generic interview guides developed for different types of respondents, to be adapted to the local context by each case study team.

The qualitative component of the common protocol focused on understanding how SRMNCAH&N intervention priorities were identified in each setting, and how and why specific implementation and delivery decisions were made by various actors at different levels of the health system. In-depth interviews focused on the influence of considerations such as data availability; insecurity; prevailing coordination mechanisms; and the availability of financing, health workers, commodities, and other resources needed to deliver health and nutrtion services. At the same time, the protocol encouraged particular attention to the differences between geographies, target populations, and health intervention domains across the continuum of care for women and children.

The quantitative component of the common protocol aimed to analyse changes in the coverage of key SRMNCAH&N interventions over time, at national and subnational levels, to help inform the collection of qualitative data and to interpret the results of their analysis. The protocol encouraged identification and utilization of all available data including national surveys, smaller scale surveys and assessments, and government, humanitarian agency or facility-specific health information systems, and other sources where possible and appropriate.

The common protocol and its instruments were then adapted by the individual research teams working in each country to ensure local acceptability and relevance, to best reflect the contextual factors and, in some cases, the research priorities that were locally important, and to account for data, security, and other constraints that varied from country to country.

Meetings were held in Dubai in November 2018 to present and discuss country-specific protocols, progress and preliminary results. These convenings included members of the BRANCH consortium, in-country research partners, and international research partners. Through these consultations, research teams were able to share cross-country learnings, discuss issues that arose during fieldwork and analysis, and adapt their research methodologies accordingly.

The articles in this collection highlight the various ways humanitarian actors negotiate the complex environments and constraints created or exacerbated by armed conflict in order to meet the health and nutritional needs of women and children affected by conflict. While standardized, the case studies all offer unique and contextually different perspectives. The case studies in South Sudan and DRC, for example, document humanitarian actors’ efforts at navigating shortages of funds and skilled workforce, and limited surveillance systems. The breadth of articles also captures the large variability in contexts where conflict occurs. Case studies in Colombia and Mali, for example, investigate the very different impacts conflict can have on existing operational health systems. The Somalia case study highlights the difficulties arising from competing funding needs for humanitarian health and longer term development in a protracted conflict setting where the federal government’s current efforts are focusing on developing a functional health system. While in Nigeria, we see that conflict may have surprisingly little impact in an area that had long suffered from poor health parameters in the pre-conflict period. Humanitarian intervention has been accompanied by important improvements in health service delivery, but it remains to be seen whether these will persist in the post-conflict period. Several of the case studies also examine governance and its implications for health service delivery such as those that arise from the fragmentation of the health system in Afghanistan, or of government authority more generally in Yemen and Syria. The collection also presents applications of different methodologies for studying conflict, such as innovative efforts to capture conflict severity by triangulating quantitative mortality data with systematic polling of implementing agencies and other local stakeholders in the Afghanistan and Pakistan case studies. Lastly, the case studies highlighted the methodological challenges of conducting research in these settings such as navigating dynamic security situations, restricted physical access to field sites, and limited survey and surveillance data.

This collection provides a snapshot of how humanitarian actors contend with conflict and the associated barriers when delivering health and nutrition services to women and children. It is our hope that these articles can impart important learnings that will advance the field of humanitarian health for women and children.

## Supplementary information

**Additional file 1.** BRANCH Country Case Study Common Protocol.

## Data Availability

N/A.
