# Supplementary material for: Investigating the delivery of health and nutrition interventions for women and children in conflict settings: a collection of case studies from the BRANCH Consortium
Source: Confl Health. 2020 May 27;14:29. doi: 10.1186/s13031-020-00276-y (PMC7254714; doi:10.1186/s13031-020-00276-y)
Supplement: Supplementary file 1 — Additional file 1. BRANCH Country Case Study Common Protocol. [file 13031_2020_276_MOESM1_ESM.pdf]

BRANCH Country Case Studies

1. Overview of Case Studies

1.1. Rationale

Conflict and disasters disproportionately affect children and women.(1-4) Best estimates of maternal mortality in conflict settings are 1,000 per 100,000 live births compared to 690 per 100,000 live births in non-conflict settings in Sub-Saharan Africa.(5) Approximately 50% of global maternal deaths occur in six countries that have suffered or are in the midst of an armed conflict.(6) Maternal and under-5 mortality are notably higher in countries with an ongoing conflict compared to their non-conflict counterparts.(5) Another study in 28 Sub-Saharan Africa found that under-5 mortality increased by 1.3% during conflict.(7)

Conflict has both direct and indirect health effects on children.(8) (9) Injury and death increase as a direct result of armed conflict.(10) The largest impact of conflict is not direct injuries due to conflict but instead malnutrition, malaria and other childhood illnesses.(9) Mortality rates among children under-5 are considerably higher in humanitarian crises due to the synergy of many factors including violence, displacement, infectious disease, poverty and malnutrition.(11) (12) As in stable settings, diarrhea, measles, malaria, pneumonia and acute malnutrition cause the majority of child deaths in humanitarian crises, however, children face the additional burden of psychological trauma as a consequence of their exposure to conflict.(12-14)

Sexual and reproductive health problems are leading causes of women's ill health and deaths worldwide. Vulnerability to these problems and the risk of mortality increase in crisis situations, especially among pregnant and postpartum women.(11) Studies in post-emergency and crisis settings show that pregnant women may have increased medical risks such as gestational hypertension and anemia, along with adverse pregnancy outcomes including low birth weight or preterm birth.(15) Humanitarian crisis further increase the risk of pregnancy-related death due to pre-existing nutritional deficiencies,(15, 16) susceptibility to infectious diseases,(2, 15, 16) lack of access to antenatal care,(15, 17-19) and the unavailability of assisted deliveries and emergency obstetric care.(2, 16, 17, 19)

Several factors worsen the health outcomes of women and children in conflict settings. Conflict disrupts access to preventative and curative health services. Conflict diverts government spending from health services to weapons and munitions,(16, 20, 21) while aerial bombing and ensuing fighting destroy health infrastructure and weaken the provision of preventive services.(16, 20-22) Conflict drives up the cost of health care as a result of limited health personnel and medical supplies.(18) Insecurity can also disrupt physical access to services, as safety concerns prohibit travel. Increased economic pressure and poverty can also place women and children in vulnerable situations as they are separated from male breadwinners who were their primary source of income and must now find other sources of economic support.(1) Forced displacement and financial constraints necessitate individuals seeking refuge in makeshift shelters. Individuals are forced to live in inadequate conditions which expose them to accident, disease, and injury.(1, 20) These shelters are overcrowded, with limited access to water, heating, and sanitation facilities.(2, 16, 21-23) Disease transmission of HIV/AIDS, cholera, tuberculosis,

and malaria increases in these inadequate living conditions.(16) Exposure to violence during the conflict can have longstanding physical and mental trauma for both women and children.(13, 14) Additionally, women may be particularly vulnerable to gender-based violence. Intimate partner violence is the most common form of gender-based violence in conflict settings but women also experience sexual violence in camp settings, or as systematic violence perpetrated by parties to the conflict.(24)

Crucial interventions including contraceptive provision,(25) antenatal care,(16, 25) skilled birth attendance,(25) clean delivery kits,(25) promotion of immediate and exclusive breast-feeding,(25) nutrition education and support,(26) and the provision of soap and avoiding contamination of water,(27) can have a major impact on morbidity and mortality in conflict settings. Providing these interventions to conflict-affected populations presents difficulties, particularly in insecure areas. Instability and fear of violence can also limit a family's ability to travel to receive health services. With a multitude of global actors working in the humanitarian space attempting to overcome the obstacles presented by conflict, it is important to consolidate this expertise. It is essential to understand sexual, reproductive, maternal, newborn, child, and adolescent health and nutrition (SRMNCAH & N) intervention delivery in conflict settings. In particular, it is important to understand the numerous factors and considerations that are underlying the decisions to use these delivery methods. Our project aims to explore the provision of SRMNCAH & N interventions in conflict settings and describe the factors that influence the implementation of SRMNCAH & N in these settings. We will conduct case studies in ten countries that have experienced conflict. We have chosen a range of different countries in order to capture multiple experiences of conflict and add depth to our analysis. Through understanding how those practicing in the field are overcoming obstacles delivering health interventions in conflict, this study can inform best practices describing how to deliver health services. Ultimately, the results from this study aim to improve care and outcomes.

2. Study Context: Case study countries

Ten countries will inform the case studies. Our country selection was influenced by the feasibility of conducting research within that context. Feasibility was characterized by security, access, data availability, and interested local research and implementing partners. The selected countries were identified to reflect a mix of geographical representation, phase of conflict, displacement scenarios, income level, and level of documentation. The case study countries include:

- Afghanistan
- Colombia
- DRC
- Mali
- Nigeria
- Pakistan
- Somalia
- South Sudan
- Syria

- Yemen

The period of investigation will differ for each country, and is informed by peaks of battle-related deaths. Battle-related deaths are being used as a marker of conflict intensity under the assumption that the period with the highest number of deaths would align with when funding and NGO activities in the country were also the highest. (see Table 1).

**Country**	**Peak BRD**	**Year BRD upward trend contributing to peak started**	**Peak BRD in last 5 years (if different)**	**Year BRD upward trend contributing to peak started (if peak BRD in last 5 years is different)**
Afghanistan	2015	2013		
Pakistan	2010	2006	2014	2013
Syria	2013	2011		
Yemen	2015	2013		
Somalia	1992	1990	2012	2011
South Sudan	2014	2012		
Mali	2013	2011		
Nigeria	2014	2009		
DRC	2013	2011		
Colombia	2002	2000		

Table 1: Trends for Battle Related Deaths (BRD)

3. Research Plan

**Component**	**Geography of Focus**	**Interventions**	**Period of Interest**	**Countries**	**Categories of Data**	**Data Analysis**
**Component 1: SRMNCAH & N coverage**	All conflict-affected populations and geographies of interest	SRMNCAH & N[a]	2000-2017	Ongoing conflict and transitioning conflict countries	Quantitative data and document review	Quantitative Analysis (as feasible due to data availability)
**Component 2: Overview of Operations**	All conflict-affected populations and geographies of interest	SRMNCAH & N	Country specific time period (see Table 1)	Ongoing conflict and transitioning conflict countries	Document review and primary qualitative data	Latent content Analysis

[a] SRMNCAH & N key interventions of interest are defined in Appendix 2

Component 1: SRMNCAH & N coverage
Geography of focus: All conflict-affected populations and geographies of interest
Interventions: SRMNCAH & N
Period of interest: 2000-2017[b] (as available)
Countries: Ongoing conflict and transitioning conflict countries
Categories of Data: Secondary data: quantitative data and document review

Component 1: Research Questions

The country case study team will focus on using existing data to determine what interventions across the continuum of care were delivered, at what scale, and the impact of interventions on morbidity or mortality in the case study country. Several questions will guide their investigation:

1. What health domains[c] (including the interventions) across the continuum of SRMNCAH & N care were addressed by
    1.1. Geography (i.e. describe patterns of distribution throughout the country)
    1.2. Time (i.e. describe if and how attention to different health domains changed over time)
    1.3. Organization (i.e. how different organizations addressed various health domains)
    1.4. Interrelationship of geography, time, and organization (i.e. describe the relationship between geography, time and organization including patterns and trends)

2. How interventions in health domains across the SRMNCAH & N continuum of care were delivered (e.g. packaged interventions, healthcare workers, types of health facilities etc.) by
    2.1. Geography (i.e. describe how service delivery differed by geographic areas)
    2.2. Time (i.e. describe how types of service delivery have changed over time)
    2.3. Organization (i.e. describe the range of service delivery modalities and how it differs among organizations)
    2.4. Interrelationship of geography, time, and organization (i.e. describe the relationship between geography, time, and organization including trends)

3. At what scale were specific interventions within each domain across the continuum of care delivered by
    3.1. Geography (i.e. describe how the scale of each intervention differed by geographic area)
    3.2. Time (i.e. describe how different interventions have been scaled up or down over time)

---

[b] 2000-2017 was chosen because it is extended enough time period within which to document trends in coverage.
[c] Domain refers to health area of investigation, i.e. sexual, reproductive health, maternal health etc. while interventions refer to the specific health service as described in Appendix 2. A health domain may have multiple interventions associated with it.

3.3. Organization (i.e. describe the scale at which different kinds of organizations deliver interventions)
3.4. Interrelationship of geography, time, and organization (i.e. describe the relationship between geography, time and organization including trends in the scale of interventions)

4. What was the effect of the SRMNCAH & N interventions on morbidity and/or mortality?

Component 1: Potential Data Sources (see section 4.1 below for more detail on data types)

- National or sub-national population surveys
    - DHS
    - MICS
    - Other household surveys including information on health, living conditions and access to services
- Government- sourced information
    - Policy and strategy documents
    - Clinical protocols
    - Health information system reports
    - Aggregated hospital/health facility data
    - CHW records/community- based program data
    - Policy reports/documents
    - Expenditure information including national health accounts and health sector budget reports
- UN and NGO- sourced information
    - Project proposals/reports/evaluations
    - Needs assessments
    - Programmatic data (including activity inputs, processes and outcomes tracked)
    - Health service statistics
    - Clinical protocols
    - Population surveys and health facility assessments
    - Expenditure information
    - Humanitarian Cluster and other health coordination meeting minutes

Component 2: Overview of Operations and Delivery
Geography of focus: All conflict-affected populations and geographies of interest
Interventions: RMNCAH & N
Period of interest: Country- specific time period (see Table 1)
Countries: Ongoing conflict and transitioning conflict countries
Categories of Data: Document review and primary qualitative data

Component 2: Research Questions

The country case study team will focus on the operations of different kinds of organizations during the period of interest. This includes decision making on resource allocation to different health domains within RMNCAH & N, and coordination with different kinds/types of organizations. Several questions will guide their investigations:

1. How was evidence used to inform the decision-making processes for prioritizing health interventions for service delivery, specifically:
   1.1. Local surveys, needs assessments, routine data sources etc.
   1.2. Global evidence base on effectiveness of interventions
   1.3. Global guidance (e.g. sphere, WHO, IASC, UNHCR, MISP etc.)

2. How are interventions being delivered?
   2.1. What health worker are used to deliver interventions?
      2.1.1. What qualifications do these health workers have? What additional training are they given?
      2.1.2. Why is this cadre of health workers used to deliver the interventions?
      2.1.3. What were challenges faced in workforce availability and technical capacity and how were these addressed?
   2.2. Where does the conflict-affected population receive each intervention (e.g. local school, home, health facility etc.)?
      2.2.1. Why was this location chosen?
   2.3. Were there special efforts to reach vulnerable populations[d]?
      2.3.1. Does the delivery approach reach the most vulnerable populations? Why or why not?
   2.4. What innovative methods are being used to access conflict-affected populations?

3. What role did the availability of resources play in the decision to deliver interventions including
   3.1. Stockpiles of commodities
   3.2. Funding limitations or restrictions, such as
      3.2.1. Liquidation of funds (i.e. how quickly funds could be accessed by country team and its role on the timing of service delivery)
      3.2.2. Budget constraints
      3.2.3. Availability of assessments of relative cost and the cost effectiveness of interventions
      3.2.4. Sources of funding (e.g. tied funding)
      3.2.5. Fluctuations in funding
   3.3. The types and numbers of local and international health workers (across different geographies in the country)
      3.3.1. What capacity-building strategies were possible? Which ones were not?

[d] Vulnerable populations are defined as members of socially marginalized groups who are discriminated against on the basis of their gender, class, caste, race, ethnicity, tribal affiliation, religious affiliation, geographic area of residence, and other local factors

4. How did country-specific contextual factors limit or facilitate which interventions were delivered?
   4.1. National laws and legislation (including reporting of specific interventions)
   4.2. Security conditions due to the conflict
      4.2.1. Did this change over time?
      4.2.2. Did this change by geographical variation?
      4.2.3. How were security concerns managed?
   4.3. Culturally-situated beliefs (i.e. describe how these influence the acceptability of different health interventions)
   4.4. Strength of public, private, and faith-based care networks
   4.5. Political dynamics (e.g. power sharing dynamics or geographic areas of control)

5. How did actors (including local and international NGOs, UN agencies, and government) coordinate with one another (bilaterally and/or within clusters) to provide SRMNCAH & N interventions?
   5.1. What means were used to ensure coordination? (e.g. cluster meetings and who participated or did not?)
   5.2. What objectives were coordination aimed at? (e.g. avoiding duplication, sourcing complementary resources etc.)

6. How did organization operations influence the selection of interventions including
   6.1. How did organizational expertise and mandate influence which interventions across the continuum of care were delivered?
   6.2. How did established institutional practices/patterns of behavior of delivery influence which interventions across the continuum of care were delivered?

7. How has increased attention to one domain within SRMNCAH & N influenced attention to another aspect of SRMNCAH & N including through distribution of:
   7.1. Commodities
   7.2. Resources
   7.3. Workforce
   7.4. Financing

8. What other enabling and constraining factors affect the delivery and implementation of interventions?

9. Has the set of interventions delivered evolved over time? Why?
   9.1. What has been the role of epidemics on the introduction of new interventions?
   9.2. What has been the role of natural disasters on the introduction of new interventions?
   9.3. How have any influxes of displaced populations influenced the introduction of new interventions?
   9.4. What other factors influenced changes?

Component 2: Potential Data Sources

- Government-sourced information
    - Policy reports/documents
    - Expenditure information
    - Memos/emails
- UN and NGO sourced information
    - Programmatic data
    - Project/Program proposals
    - Project/Program reports
    - Project/Program evaluations
    - Minutes of meetings
    - Annual reports
    - Expenditure information
    - Sitreps (report on the military situation in a particular area)
    - Memos/emails
    - Cluster and other health coordination meeting minutes
    - FTS (Financial Tracking Service)
- Media
    - Newspaper articles
- Key informant interviews and focus group discussions
    - NGO upper management (i.e. country representative/medical coordinator/logistic coordinator/security coordinator)
    - NGO healthcare staff (i.e. site coordinator, frontline fieldworker/clinicians, program coordinators)
    - Government and Opposition Forces (e.g. Ministry of Health and/or other equivalent governing bodies, provincial and district level health authorities)
    - UN officials (i.e. representatives from UNICEF, WHO, UNHCR, and/or UNFPA)
    - Health facility staff (including management and clinicians)
    - Additional as appropriate

4 Methods

4.1 Types of Data

Several different types of data will be used to inform the data collection, they are described below. The country case study lead and team will decide which data sources will be used to inform the research objectives from the potential data sources listed for that component. They will decide how to best address the research objectives given the availability of secondary data, and documents in the country. The potential data sources are described in detail below.

	Component 1-Quantitative	Component 2 –Qualitative

**Data sources**	• National or sub-national population surveys<br>o DHS<br>o MICS<br>o Other household surveys including information on health, living conditions and access to services, monitoring and evaluation findings<br>• Government- sourced information<br>o Policy and strategy documents<br>o Clinical protocols<br>o Health information system reports<br>o Aggregated hospital/health facility data<br>o CHW records/community-based program data<br>o Policy reports/documents<br>o Expenditure information including national health accounts and health sector budget reports<br>• UN and NGO- sourced information<br>o Project proposals/reports/evaluations<br>o Needs assessments<br>o Programmatic data (including activity inputs, processes and outcomes tracked)<br>o Health service statistics<br>o Clinical protocols<br>o Population surveys and health facility assessments<br>o Expenditure information<br>o Humanitarian Cluster and other health coordination meeting minutes	• Government-sourced information<br>o Policy reports/documents<br>o Expenditure information<br>o Memos/emails<br>• UN and NGO sourced information<br>o Programmatic data<br>o Project/Program proposals<br>o Project/Program reports<br>o Project/Program evaluations<br>o Minutes of meetings<br>o Annual reports<br>o Expenditure information<br>o Sitreps (report on the military situation in a particular area)<br>o Memos/emails<br>o Cluster and other health coordination meeting minutes<br>o FTS (Financial Tracking Service)<br>• Media<br>o Newspaper articles<br>• Key informant interviews and focus group discussions<br>o NGO upper management (i.e. country representative/medical coordinator/logistic coordinator/security coordinator)<br>o NGO healthcare staff (i.e. site coordinator, frontline fieldworker/clinicians, program coordinators)<br>o Government and Opposition Forces (e.g. Ministry of Health and/or other equivalent governing bodies, provincial and district level health authorities)<br>o UN officials (i.e. representatives from UNICEF, WHO, UNHCR, and/or UNFPA)<br>o Health facility staff (including management and clinicians)<br>o Additional as appropriate

4.1.1 Literature Review

An in-depth literature review on the case study country will be conducted prior to the data collection process. The literature review will provide information on the details and nature of the conflict, and the status of the health domains across the continuum of care. The country case study lead and team are responsible for compiling any literature they believe is required prior to commencing the data collection. The literature review will include the scientific and grey literature. The grey literature will include published documents and reports on the study settings from non-profit governmental organizations, government hospitals, and intergovernmental

organizations. These will be procured using online databases, direct contact with the organizations, and archives.

A systematic review on the health domains across the continuum of care is currently being conducted, however, the results from the systematic review will not be available until the summer of 2018. The compiled results will be shared with the case study leads. Additionally, as the systematic review is being conducted, any article on a case study country is tagged and placed in a separate folder regardless of whether they are included within the systematic review. Once completed, these articles will also be shared with the case study leads. The literature procured through the systematic review can be used to contextualize the results that emerge from the case study.

A systematic review on sexual and reproductive health in conflict is currently being completed by the team from LSHTM. The results from this review will be available to the co-investigators in November 2017.

4.1.2 Document Review

Documents will be obtained from partner organizations and may include internal communications such as memos, emails, and minutes from meetings. Policy documents, project/program proposals, project/program reports, project/program evaluations, clinical protocols, sitreps, expenditure information, and any other additional documents will also be reviewed. Some documents will be sourced from government agencies and include expenditure information, memos, and emails. UN sourced data such as cluster meeting minutes, and country reports will be included as will other data sources include the financial tracking service. Document review will be used as data triangulation and theory triangulation,(28) and also help raise new research questions and identify new informants. Together, these documents will be used to understand the decision-making processes that determined which interventions were delivered and their delivery methods. A potential problem related to this data source is the issue of reporting bias. Documents may reflect the biases, unbeknownst to the reader, of those who wrote or produced these documents. (29) In order to overcome this problem, the case study team will check the validity of information published in reports during interviews.

4.1.3 Quantitative Data

Quantitative data will be gathered from several data sources including the DHS, MICS, national risk and vulnerability assessment, household surveys, immunization data, hospital/health facility records, CHW records/community based program records, programmatic data (including doses, commodities delivered, health sessions, health centers supported etc.), SMART surveys, and any other local relevant surveys.

The country case study team will assess the availability of quantitative data in respect to the research objectives and use the data sources they deem most appropriate to address these objectives. For instance, in a country where there are several national surveys available, the focus on programmatic data may be minor. In contrast, in a country where there are few, outdated or spotty national figures available, programmatic data or smaller scale surveys may be needed to address the research objectives.

4.1.4 Primary qualitative data

4.1.4.1 Participants

Several different categories of participants may be included in the country case study. The country case study team will determine which categories of participants to interview depending on who can best provide the primary qualitative data required to fulfil the research objectives. These categories include:

- NGO upper management (i.e. country representative/medical coordinator/logistic coordinator/security coordinator)
- NGO healthcare staff (i.e. site coordinator, frontline fieldworker/clinicians, program coordinators)
- Government and Opposition Forces (e.g. Ministry of Health and/or other equivalent governing bodies, provincial and district level health authorities)
- UN officials (i.e. representatives from UNICEF, WHO, UNHCR, and/or UNFPA)
- Health facility staff (including management and clinicians)

It is estimated that 35-55 key informant interviews will be needed to address our study objectives by capturing the experiences of the breadth of different actors across different geographies (see figure 1). The following categories and figures are meant to serve as a guide for sampling, and may vary in different countries, especially as a reflection of the number of geographic areas of focus selected for the case study work. Country case study teams are expected to adapt the distribution of interviews in response to the number and types of actors active in the case study country. For instance, in a country where there are only one or two UN agencies actively involved only four interviews may be needed with that category of informant, while more interviews may be needed with NGO staff. If possible, interviews will be conducted until theoretical saturation.

National Representatives (n=~17-27)

UN agencies (n=~5)	Governing Entity (i.e. government or opposition) (n=~2)	International NGOs (n=~5-10)	Local/National NGOs (n=~5-10)
• UNICEF<br>• UNHCR<br>• UNFPA<br>• WFP<br>• WHO	• Federal official	Upper management (leadership and program management or technical leads (plus logistics, finance as applicable	• Upper management (leadership and program management or technical leads (plus logistics, finance as applicable)

Sub-national/Regional Representatives (n=~18-28)

UN agencies (e.g. field offices) (n=~5)	Governing Entity (i.e. government or opposition) (n=~3)	International NGOs (n=~5-10)	Local/National NGOs (n=~5-10)
• UNICEF<br>• UNHCR<br>• UNFPA<br>• WFP<br>• WHO	• Regional or District level official	• Program management or technical leads<br>• Point of Care staff[1]	• Program management or technical leads<br>• Point of Care staff[1]

Total: n=~35-55

Figure 1: Participant sample size

A purposive, rather than a probabilistic sampling method will be deliberately used by the investigator.(30, 31) Purposive sampling is used when researchers "seek out groups, settings and individuals where … the processes being studied are most likely to occur".(28)

The snowball technique will be applied to identify individuals who can best speak to our research objectives. At the end of interviews, the researcher will ask the interviewee to identify people who could provide valuable information.(30) As explained by Patton, (32) "the chain of recommended informants would typically diverge initially as many possible sources are recommended, then converge as a few names get mentioned over and over". This technique proved to be particularly helpful in identifying individuals working in other facilities or social sectors such as education or protection.

The snowball technique is susceptible to selection bias as interviewees tend to mention people who have the same opinions as theirs.(33) Purposeful sampling methods carry the potential risk of orientating research results towards a single direction influenced by the small size of the sample and the selectivity of those who are chosen for interview.(32) In order to balance these concerns, other sources of evidence will be used to corroborate any insight by such informants and to search for contrary evidence as carefully as possible.

4.1.4.2 Direct and participant observation

Direct and participant observation will supplement the quantitative analysis, document review, and key informant interviews. Direct observation will provide the advantage of being appropriate for studying the behaviour of and interactions between staff themselves. Direct observation will be conducted through visits to clinics and IDP/refugee camps, and more formal activities such as observation of management meetings. The focus of the observation during management meetings will be on the content of the discussions and how solutions to a specific issue will be identified or determined by the group. The country case study team will also pay special attention to the physical spaces they frequent including the physical geography (e.g. terrain, climate, ease of access roads), the facilities (e.g. cleanliness, organization, atmosphere, layout), sociocultural context (e.g. gendered mobility), and any other information relevant to the research objectives.

As recommended by Stake,(34) the investigator will concentrate on a specific set of events and actors during direct observation, which will help with:

- mapping out the actors involved in the field, as well as familiarising the investigator with formal structures and processes within the health and humanitarian sector;
- gathering information in relation to the facts and events, the nature and processes of interaction of the different partners, and their behaviour and views in relation to events;
- providing insight about the behaviours of the parties by throwing light on or challenging statements made during interviews;
- identifying individuals who should be interviewed by observing individuals during meetings and noting the type of information they dismiss or the views they express; and
- obtaining documents shared amongst participants (often evaluation reports or progress reports).

Field notes of observations and conversations between different parties will be carefully recorded.(35)

A potential bias in the direct observation method is that actors may change their attitude in the presence of the observer.(31, 36) A comparison between the observation notes and the minutes of previous management meetings will show whether the presence of the observer will influence the content of discussions.

The country case study team will use direct and participant observation to generate a narrative description within which to contextualize their findings from the quantitative analysis, document review, and key informant interviews. It will also highlight possible issues to probe in later interviews. Due to ethical concerns, participant observation will not include observations of direct care provision to recipients of interventions.

4.1.4.3 Interviews

According to Stake, “the interview is the main road to multiple realities.”(34) Capturing the various perspectives of one reality forms the ultimate goal of qualitative research and case studies.(34) In-depth interviews tend to be characterized by “their flexible and interactive nature, their ability to achieve depth, the generative nature of data … in the sense that new knowledge or thoughts are likely, at some stage, to be created.”(37) Interviews will be conducted with the aforementioned participants as deemed appropriate by the country case study team.

Interviews will follow a semi-structured interview guide with each interview lasting approximately one-two hours (see Appendix 1). Semi-structured interviews have the advantage of ensuring that the respondent has a good understanding of the topic discussed, and that sensitive and complex issues can be broached.(38) Interview guidelines will be elaborated and

adjusted according to the interviewer's requirements.(39) The interviewer will respond to the respondent's knowledge and ability to speak to the study objectives. The semi-structured guide will serve as a guide, and the interviewer will use to emphasize areas that will garner most meaningful information from the respondents.

Recall bias may present a problem during the data collection process. In order to compensate for this potential bias, documentary analysis will be used to identify the events that had occurred at the beginning of the intervention.

4.1.4.4 Focus Group Discussions

If deemed appropriate the case country study team can opt to conduct focus groups with their respondents. These may include staff from the health facility, and/or from a variety of NGOs. The focus group guide will be created by the country-specific case study team and based upon the interview guide (see Appendix 1). Through the focus groups, the research team will aim to understand any gaps in service provision. During the focus groups, special attention will be paid to interactions between various NGOs to understand the working dynamics between groups.

5 Data Analysis

Our project is a mixed methods case study which aims to fulfil our research objectives (section 3) through providing a rich description of how SRMNCAH & N interventions are delivered in conflict settings, in particular, and the reasons underlying the decisions and numerous factors that contribute to these delivery methods. The analytical techniques reflect these aims.

5.1 Quantitative Data Analysis

Secondary analyses will be conducted on the available quantitative data on intervention coverage (see Appendix 2) and/or on morbidity and mortality in the study population over the study period. Where possible, quantitative data will be obtained from national surveys, NGOs, and UN agency partners and collaborators. These data will thus include programme as well as survey and routine data. Descriptive analyses will focus on trends in intervention coverage and, if possible, patterns and trends in morbidity and mortality (see research objectives section 3). The quantitative analytical plan will be country specific as a reflection of data availability in the country.

5.2 Qualitative Data Analysis

Latent content analysis will be used to analyze the data.(40) The iterative nature of qualitative research dictates that there is overlap between the data collection and analysis. When possible, after the first interview is completed, the recording or notes (if the interviewer has declined recording) will be sent immediately for transcription and translation so that analysis can begin as soon as possible. Daily debriefing sessions with the field research team, and frequent meetings with other members of the country case study team will also highlight other possible

areas of inquiry. Any new areas of investigation will be probed with respondents, and shared with other consortium countries so they can explore these areas of investigation in their own work.

Our data analysis will be guided by our research objectives. The analysis requires specific methods that are dependent upon the source of data:

1. Interviews
    - Interview data will be analyzed using latent content analysis
    - Data will be manually coded but transcripts will be managed in software of the country case study team’s preference (e.g. NVivo, Atlas ti etc.)

2. Notes from observations of management meetings
    - Notes are less structured than interview transcripts and their content is less predictable than interviews, however, a similar coding system can be used.
    - The new themes identified in the notes will be translated into codes.

3. Document review
    - All documents will be indexed and key documents will be summarized as determined by the country teams in the country specific protocol
    - The results from the document review will be included into the analysis as a complementary source of data to add evidence to elements of information collected through observation or interviews.

4. Field notes
    - Field notes are of crucial importance during preliminary analysis of data
    - Field notes, in particular analytic notes, will be read once again during the final analysis process in order to ensure that all the themes identified during data collection are covered by the coding system.

Once the country- specific work has been completed, further analysis of the results across the case studies will be conducted. The results from the country- specific case studies will be integrated.

6. Rigor: Qualitative Data

Rigor in qualitative research is focused on defining the trustworthiness of the research. Lincoln and Guba first tackled the issue proposing the criteria credibility, fittingness, and auditability be used to assess trustworthiness.(40) Lincoln and Guba later refined the concept of trustworthiness so it included credibility, transferability, dependability, and conformability.(40)

Unlike in quantitative research, which is primarily concerned with validity and reliability, qualitative inquiry assumes the results the researcher draws within the study are context specific,(41) and if another individual were given the same data, they may analyze them in a

different way.(40, 42) Rigor in qualitative research, therefore, is concerned with conducting clear and thorough analysis.

Several methods will be used to ensure the rigor of our primary qualitative data collection and analysis methods. An audit trail will be created by the case country lead which will document the data collection and analysis process.(40) The audit trail will describe the decision-making process, including what informed these decisions. The audit trail will include a reflexive journal that the researchers will maintain throughout the research process. Within this journal the researchers will describe the assumptions underlying their work. The researchers will be reflexive about the role of their epistemology, ontology, and theoretical orientation on the data analysis process. The journal will also document the decisions that were made during data collection, for instance, why one individual at one organization was interviewed instead of another, or during the analysis, it might explain how a specific category emerged. Without documentation the rationale underlying these decisions might be forgotten, and defending these analytical decisions may be difficult.(40) Peer debriefing during the analysis process will contribute to the interpretative accuracy of the data and unearth any implicit assumptions underlying the data analysis. Peer debriefing will occur within the in-country team, with the consortium member institution, and across the consortium.

Triangulation of data sources will also be used to avoid presenting partial views on a specific situation (31). One may realize that different data sources are not always in consonance with each other (28). However, as Hammersley and Atkinson (31) pointed out, it is naïve to think that data from different sources will systematically aggregate to one coherent piece of information. It is recommended that the researcher remains attentive to the context from which the data comes in order to avoid the error of aggregating different pieces of information.(43)

If possible, respondent validation will be used once the preliminary data analysis is completed. The researchers will share a document with participants that describes the preliminary results. The participants will have the opportunity to verify that the researcher has accurately interpreted their intent.

7. Ethics

Several ethical concerns may arise through our research as we are studying particularly vulnerable populations, and the research team will undertake several measures to ensure that these are addressed through our data collection and analysis process. First, informed consent will be sought from participants at the outset, and the researchers will protect the privacy and confidentiality of the respondents throughout the research process, as well as after. Respondents will be asked for permission to record the interview. If permission is not granted, the interviewer will take notes. Respondent identifiers will be removed from the interview recording, transcript, or notes. Each respondent will be assigned a number, only this number will be associated with their data. The key with the respondent’s name will be kept in a password-protected file that only the case country team will have access to. If at any point during the interview the respondent demonstrates discomfort, the interview will be halted.

Ethics approval will be sought for all case studies from the SickKids Research Ethics Board. For each individual case study, both the consortium lead (if not SickKids) and the in-country lead will additionally seek approval from their own institutions’ ethics review

committees. Where no national or academic ethics review committee is available in-country (e.g., Syria), other independent ethics review committees (e.g., MSF) will be consulted.

8. Research Team Roles and Responsibilities

Each case study will be led by one or more consortium members. The consortium member institutions will be responsible for coordinating with potential case study partners including local academic partners, NGOs, UN agencies, and/or study funders (figure 2). The constellation of partners will differ depending on the case study context.

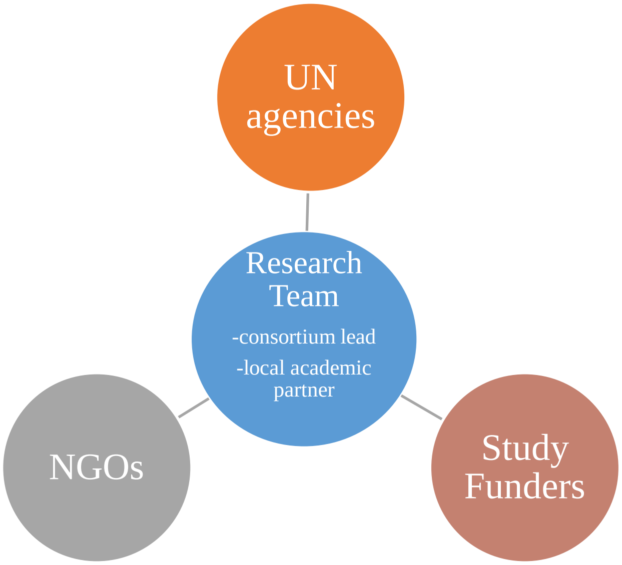

Figure 2 Potential Case Study Partners

An in-country study team will be responsible for collecting data; this team will be supervised by a representative from the consortium member institution in cooperation with the head of the in-country research team. The country case study lead will train the in-country study team on their country-specific protocol, including data collection techniques and tools and the study objectives. The in-country team will ideally have experience collecting qualitative data. In the situations where this is not the case, the country case study lead will conduct additional training on qualitative methods.

Data analysis will be led by the consortium member institution with the head of the in-country research team. The iterative nature of qualitative research means results will emerge throughout the data collection process. These results will be shared with the other consortium institutions so similar areas can be probed across case study countries.

References

1. Gardam JG, Charlesworth H. Protection of women in armed conflict. Human Rights Quarterly. 2000;22(1):148-66.

2. Gasseer NA, Dresden E, Keeney GB, Warren N. Status of women and infants in complex humanitarian emergencies. Journal of midwifery & women's health. 2004;49(S1):7-13.
3. Neumayer E, Plümper T. The gendered nature of natural disasters: The impact of catastrophic events on the gender gap in life expectancy, 1981–2002. Annals of the Association of American Geographers. 2007;97(3):551-66.
4. Bhutta ZA, Black RE. Global maternal, newborn, and child health—so near and yet so far. New England Journal of Medicine. 2013;369(23):2226-35.
5. O'hare BAM, Southall DP. First do no harm: The impact of recent armed conflict on maternal and child health in Sub-Saharan Africa. Journal of the Royal Society of Medicine. 2007;100(12):564-70.
6. Hogan MC, Foreman KJ, Naghavi M, Ahn SY, Wang M, Makela SM, et al. Maternal mortality for 181 countries, 1980–2008: a systematic analysis of progress towards Millennium Development Goal 5. The lancet. 2010;375(9726):1609-23.
7. Burke M, Heft-Neal S, Bendavid E. Sources of variation in under-5 mortality across sub-Saharan Africa: a spatial analysis. The Lancet Global Health. 2016;4(12):e936-e45.
8. Devkota B, van Teijlingen ER. Understanding effects of armed conflict on health outcomes: the case of Nepal. Conflict and Health. 2010;4(1):20.
9. Leaning J, Guha-Sapir D. Natural disasters, armed conflict, and public health. New England journal of medicine. 2013;369(19):1836-42.
10. Garfield R, editor. The epidemiology of war. New York: Oxford University Press; 2008.
11. Coghlan B, Brennan RJ, Ngoy P, Dofara D, Otto B, Clements M, et al. Mortality in the Democratic Republic of Congo: a nationwide survey. The Lancet. 2006;367(9504):44-51.
12. STC. State of the World's Mothers 2014:

Saving Mothers and Children in Humanitarian Crises. Westport, Connecticut; 2014.
13. Cabrol J-C. War, drought, malnutrition, measles—a report from Somalia. New England Journal of Medicine. 2011;365(20):1856-8.
14. Slone M, Mann S. Effects of war, terrorism and armed conflict on young children: a systematic review. Child Psychiatry & Human Development. 2016;47(6):950-65.
15. Moreno-Walton L, Koenig K. Disaster Resilience: Addressing Gender Disparities. World Medical & Health Policy. 2016;8(1):46-57.
16. Urdal H, Che CP. War and gender inequalities in health: the impact of armed conflict on fertility and maternal mortality. International Interactions. 2013;39(4):489-510.
17. Bosmans M, Nasser D, Khammash U, Claeys P, Temmerman M. Palestinian women's sexual and reproductive health rights in a longstanding humanitarian crisis. Reproductive health matters. 2008;16(31):103-11.
18. Price JI, Bohara AK. Maternal health care amid political unrest: the effect of armed conflict on antenatal care utilization in Nepal. Health policy and planning. 2013;28(3):309-19.
19. Chi PC, Bulage P, Urdal H, Sundby J. Perceptions of the effects of armed conflict on maternal and reproductive health services and outcomes in Burundi and Northern Uganda: a qualitative study. BMC international health and human rights. 2015;15(1):7.
20. d'Harcourt E, Purdin S. Impact of wars and conflict on maternal and child health. In: Ehiri J, editor. Maternal and Child Health: Springer; 2009. p. 121-33.
21. Ghobarah HA, Huth P, Russett B. The post-war public health effects of civil conflict. Social science & medicine. 2004;59(4):869-84.
22. Iqbal Z. Health and human security: The public health impact of violent conflict. International Studies Quarterly. 2006;50(3):631-49.
23. Banatvala N, Zwi AB. Conflict and health: public health and humanitarian interventions: developing the evidence base. British Medical Journal. 2000;321(7253):101.
24. Stark L, Ager A. A Systematic Review of Prevalence Studies of Gender-Based Violence in Complex Emergencies. Trauma, Violence, & Abuse. 2011;12(3):127-34.

25. Campbell OM, Graham WJ, group LMSSs. Strategies for reducing maternal mortality: getting on with what works. The lancet. 2006;368(9543):1284-99.
26. Girard AW, Olude O. Nutrition education and counselling provided during pregnancy: effects on maternal, neonatal and child health outcomes. Paediatric and perinatal epidemiology. 2012;26(s1):191-204.
27. Benova L, Cumming O, Campbell OM. Systematic review and meta-analysis: association between water and sanitation environment and maternal mortality. Tropical medicine & international health. 2014;19(4):368-87.
28. Denzin N. Sociological methods: A sourcebook. New York: McGraw-Hill; 1978.
29. Yin R. Case study research: design and methods. London: Sage Publications; 2003.
30. Bowling A, Ebrahim S. Handbook of health research methods. Maidenhead: Open University Press; 2005.
31. Patton M. Enhancing the quality and credibility of qualitative analysis. Health Services Research. 1999;34(5 Pt 2):1189-208.
32. Patton M. Qualitative research and evaluation methods. Third ed. London: Sage Publications; 2002.
33. Browne J. Survey design. In: Green J, Browne J, editors. Principles of social research. Maidenhead: Open University Press; 2006.
34. Stake RE. The art of case study research. London: Sage Publications; 1995.
35. Fitzpatrick R, Boulton M. Qualitative methods for assessing health care. Quality in Health. 1994;3:107-13.
36. Allen P. Legal and economic analysis of contracts in the NHS internal market. London: London School of Hygiene and Tropical Medicine; 2000.
37. Legard R, Keegan J, Ward K. In-depth interviews. In: Ritchie J, Lewis J, editors. Qualitative research practice: A guide for social science students and researchers. London Sage; 2003. p. 138-69.
38. Fielding N. Qualitative interviewing. In: Gilbert N, editor. Researching social life. London: Sage Publications; 1993.
39. Robson C. Real world research: a resource for social scientist and practitioner-researchers. Oxford: Blackwell Publishers; 2002.
40. Mayan MJ. Essentials of qualitative inquiry. Walnut Creek: Left Cost Press; 2009. 171 p.
41. Wheeldon J. Mapping mixed methods research: Methods, measures, and meaning. Journal of Mixed Methods Research. 2010;4(2):87-102.
42. Bergman MM. On concepts and paradigms in mixed methods research. Journal of Mixed Methods Research. 2010;4(3):171.
43. Hammersley M, Atkinson P. Ethnography: Principles in practice. London: Tavistock; 1983.

.

Interview Guide:
Point of care and/or health facility staff

I: Thank you for sitting down with me today: as you know, we are interested in understanding Sexual, Reproductive, Maternal, Newborn, Child, Adolescent Health & Nutrition intervention delivery in conflict. We know conflict is a particularly difficult context to work in, and our effort is focused on understanding how you deliver health services in these difficult contexts. We are speaking to a range of individuals involved in health service provision during conflict including different levels of NGO staff, UN officials, and government officials. In [country] we are focusing on the period between [country specific time period]. Before we begin I have a consent form that I would like you to sign. You can take as much time as you need to read over the form but I wanted to highlight a few items. First, ensuring your privacy is important to us, and we will remove all personal identifiers from our data. All recordings and notes will be assigned a code so that they are not linked to your name. The key to the codes will be kept in a password protected file which only the research staff can access. We will not link your name, or your position at your organization with your data in any of our documentation. Second, if at any time during the interview you feel uncomfortable and would like to halt the interview please let me know. I will give you a moment to look over the form, feel free to ask me any questions. When you feel comfortable doing so please sign the form. Let me know if you have any questions about the form.

give respondent time to sign the form, once form has been signed

I: This interview is being audio recorded. This is optional. If you do not want to be recorded, let me know and I will take detailed notes instead. This recording is for our records, and will only be shared with the research team. Do I have permission to record the interview?

if agrees put on recorder

if does not agree
I: That's okay I can take written notes instead, do let me know if you change your mind at any point.

I: Do you have any questions before we begin?

General Objective	Detailed objectives	Interview Questions	Probes

1. *Objective: To establish rapport and learn about participant's work experience*	To understand participant's role in [NGO/facility] including<br>• Position within hierarchy (i.e. who they report to, and who reports to them)<br>• Responsibilities (e.g. allocation of finances, resources, etc.)<br>• Geographic areas they are responsible for	**1.1 Can you explain to me what your role as [position] with [NGO/facility] entails?**	• How do you interact with<br>o [NGO]'s regional/provincial/district offices?<br>o [NGO]'s head office?<br>• Can you describe your role<br>o in the allocation of finances?<br>o the allocation of resources and commodities?<br>o in determining what geographic areas [NGO] works in?<br>o planning RMNCAH & N interventions?<br>o delivering RMNCAH & N interventions?<br>• Can you explain what geographic areas you are responsible for in your position as [role]?<br>• How are you connected to the existing health system?<br>o Are you a part of an existing host country health system or do you consider yourself separate

			from the health services that already exist?
	To build rapport and understand the participant's general experience working in conflict in [country]	**1.2 We understand that working in [country] can be quite difficult given the ongoing conflict, can you elaborate on how the ongoing conflict has affected you and your work?**	• Can you describe<br>o how you and your team feel working in [current location]?<br>o any personal precautions you have taken as a result of the conflict?
	To learn about participant's work experience including<br>• their experience in conflict<br>• their experience in their current position	**1.3 Can you describe your experience working in conflict/[country] before entering the position of [role]?**	
	To learn about participant's educational background	**1.4 Can you describe your educational background?**	
2 *Objective: To understand what interventions are delivered by [*NGO/facility*]*	To identify what interventions were delivered	**2.1 We have been looking at [NGO/facility]'s activities in [country] and have made a list of the services or programs that [NGO/facility] delivers. Could I share this list with you. [Share list of services]. Is there anything missing from the list or anything listed that is no longer delivered?**	

	To identify any gaps across the continuum of care	**2.2 So we have discussed [list services or interventions] as part of the services you provide, if you don't mind I'm going to list a few other areas of health, could you tell me if your organization provides any services or interventions related to that area? [list continuum of care areas that weren't listed in 2.1 or found in document review, probe a few examples if they say no interventions, however, do not probe on services that are outside of an organizations mandate] [ensure that you ask about breastfeeding promotion and IYCF]**	• Can you explain what considerations factored into the decision not to provide [ ] service?<br><br>Breastfeeding and IYCF probes, please inquire if there were any:<br><br>• interventions supporting milk expression?<br>• facilities where mothers can store milk?<br>• interventions supporting non-breast fed children?<br>o E.g. Wet-nursing or informal milk sharing?<br>• interventions to support complementary feeding of children from 6 to 24 months?<br>• interventions to support micronutrient supplementation of children from 6 to 24 months?
3. *Objective: To understand how interventions (across RMNCAH&N) are delivered*	To identify geographic differentials in service delivery	**3.1 Can you describe what interventions are delivered in which geographic areas?**	• Are different interventions delivered in different geographic areas? Can you describe why and what underlies these differences?

Conceptual framework areas of investigation:<br>• Health workforce	To identify geographic differentials in delivery methods	**3.2 Were there any regional differences in delivery methods of the same intervention?**	• Were different delivery methods used for<br>o different interventions in the same region? Can you explain what is underlying this difference?<br>o the same interventions in different regions? Can you explain what is underlying this difference?<br>• Can you take me through the process through which you identify the best way to deliver interventions in that area? What do you consider?<br>• Were different health workers used for<br>o different interventions in the same region? Can you explain what is underlying this difference?<br>o the same interventions in different regions? Can you explain what is underlying this difference?<br>• Can you take me through the process through which you identify the best health worker to

			deliver interventions in that area? What do you consider?
	To understand the obstacles faced when delivering interventions	**3.3 Can you describe obstacles you faced delivering this intervention? Can you give me examples?**<br><br>**3.4 Can you describe any innovative approaches that were used to overcome these obstacles?**	• Did any of the obstacles you faced require revisiting the planned delivery method?<br><br>• Were any other modes of delivery considered but eliminated as not feasible? Can you give me examples?
	To determine where individuals accessed interventions	**3.5 Can you describe where recipients access your interventions?**	• Can you describe the considerations that led to that location being identified as the place of delivery?<br><br>• How did the use of this location assist in the delivery of the intervention?<br><br>• How did the use of this location act as barrier to the delivery of the intervention?
	To determine if interventions were packaged together	**3.6 Were multiple interventions packaged together?**	
	To describe the recipients of interventions	**3.7 Who accessed your interventions? Can you describe this group?**	• Who were the intended recipients of interventions?

		**(Gender? Age? Educational status? Refugee and/or IDPs? Camp residents and/or dispersed population? etc)**	• Were there any regional variations in the type of recipient who accessed interventions?<br>• Were there differences between intended recipients and actual recipients? If so, why do you think that is?<br>• Were there any groups that this delivery method did not work well for? Can you describe this group?<br>• Were there any special efforts to try to access this group? Can you give an example?
4. Objective: To understand the contextual factors influencing the delivery of health interventions<br><br>Conceptual framework areas of investigation:<br>• Security context<br>• Sociocultural context	To unpack the impact of insecurity and conflict	**4.1 Can you briefly overview the current security situation in [country]? (e.g. access, incidents)**<br><br>**4.2 Can you describe the impact of security constraints on your work? Can you give me some examples?**<br><br>**4.3 Can you describe how your team managed security concerns?**	• Were visas or special permissions required for personnel entrance into the country or for the deployment of commodities, medications, or equipment?<br>o From whom were permissions sought?<br>• Were permissions required for the deployment of facilities or service providers?<br>o From whom were permissions sought?<br>• To what extent were security considerations a basis for

			confining services to specific locations, such as IDP or refugee camp settings?<br><br>• Did the host government influence the location of health facilities or services?<br><br>• With whom did the providers of services negotiate security?<br><br>• Did security forces accompany the transport of personnel, commodities, or equipment to service locations?<br><br>• What groups provided security for health facilities or providers of services?<br><br>• Have security forces<br>o influenced where or what services were provided?<br>o influenced which populations or patients were to be provided services?<br>o screened recipients of services before or at the service facility?

			o interfered with the provision of services?<br><br>• Have any humanitarian workers or facilities been victims of violence or received threats of violence? If so, from whom (if known)?<br><br>• Were non-standard fees or covert payments required to operate facilities?<br><br>• Was there any coordination with any opposition forces/tribal councils/other groups/country-specific category to allow the delivery of your intervention?

	To unpack the role of the sociocultural context on the delivery of interventions	**4.4 Can you explain how the sociocultural context (e.g. gender, caste, ethnicity etc.) influenced intervention selection/prioritization or delivery? Can you give me an example?**	• Were there any subpopulations that were particularly hard to access? Can you describe this group?<br>• Were there any special efforts to try to access this subpopulations? Can you give an example?<br>• Were different delivery methods used for different services in the same region? Can you explain what is underlying this difference?<br>• Did the sociocultural context affect service provision in one health domain in particular? Can you give me an example?<br>• (context specific probes will need to be generated by each country team)
	To describe any other enabling and constraining factors not mentioned	**4.5 Can you describe any other factors that affected the delivery or implementation of interventions?**	
5. Objective: To understand changes in the set of interventions delivered<br><br>Conceptual framework areas of investigation:<br>• Finances<br>• Health workforce	To understand how the provided interventions have changed paying particular attention to the impact of:<br>• Funding changes<br>• Changes in security context	**5.1 Were there any changes to the services that were planned compared to those that were delivered?**<br><br>**5.2 Can you describe how these services changed?**	• How did fluctuations in the level of funding affect service provision?<br>• Can you describe the impact of the changing security context on service provision?

• Commodities and resources<br>• Security context		**5.3 What factors influenced changes?**	• Were any formal or informal evaluations conducted? Did these indicate the need for a change in the services you provide? Could you give me an example?<br><br>• Were there any substantial influxes of displaced populations?<br>o Can you describe how this influx influenced your service provision? Can you give me an example?<br>o Did [NGO/facility] receive additional funds to provide services to the influx of population?<br><br>• Were there any epidemics while you were in the field?<br>o How did this influence your service provision? Can you give me an example?<br>o Did [NGO/facility] receive additional funds to address the epidemic?<br>o Can you describe how long the effects of the epidemic were felt?

			o Can you describe what happened with health services once the epidemic was addressed?<br><br>• Were there any natural disasters while you were in the field?<br>o How did this influence your service provision? Can you give me an example?<br>o Did [NGO/facility] receive additional funds to address the impact of natural disaster?<br>o Can you describe how long the effects of the natural disaster were felt?<br>o Can you describe what happened with health services once the situation with the natural disaster was resolved?

Interview Guide:
UN official

I: Thank you for sitting down with me today: as you know, we are interested in understanding Sexual, Reproductive, Maternal, Newborn, Child, Adolescent Health & Nutrition intervention delivery in conflict. We know conflict is a particularly difficult context to work in, and our effort is focused on understanding how you deliver health services in these difficult contexts. We are speaking to a range of individuals involved in health service provision during conflict including different levels of NGO staff, UN officials, and government officials. In [country] we are focusing on the period between [country specific time period]. Before we begin I have a consent form that I would like you to sign. You can take as much time as you need to read over the form but I wanted to highlight a few items. First, ensuring your privacy is important to us, and we will remove all personal identifiers from our data. All recordings and

notes will be assigned a code so that they are not linked to your name. The key to the codes will be kept in a password protected file which only the research staff can access. We will not link your name, or your position at your organization with your data in any of our documentation. Second, if at any time during the interview you feel uncomfortable and would like to halt the interview please let me know. I will give you a moment to look over the form, feel free to ask me any questions. When you feel comfortable doing so please sign the form. Let me know if you have any questions about the form.

give respondent time to sign the form, once form has been signed

I: This interview is being audio recorded. This is optional. If you do not want to be recorded, let me know and I will take detailed notes instead. This recording is for our records, and will only be shared with the research team. Do I have permission to record the interview?

if agrees put on recorder

if does not agree

I: That's okay I can take written notes instead, do let me know if you change your mind at any point.

I: Do you have any questions before we begin?

General Objective	Detailed objectives	Interview Questions	Probes

2. *Objective: To establish rapport and learn about participant's work experience*	To understand participant's role in [UN agency] including<br>• Position within hierarchy (i.e. who they report to, and who reports to them)<br>• Responsibilities (e.g. allocation of finances, resources, etc.)<br>• Geographic areas they are responsible for	**1.1 Can you explain to me what your role as [position] with [UN agency] entails?**	• How do you interact with<br>o [UN agency]'s regional/provincial/district offices?<br>o [UN agency]'s head office?<br>• Can you describe your role<br>o in the allocation of finances?<br>o the allocation of resources and commodities?<br>o in determining what geographic areas [UN agency] works in?<br>• Can you explain what geographic areas you are responsible for in your position as [role]?
	To build rapport and understand the participant's general experience working in conflict in [country]	**1.2 We understand that working in [country] can be quite difficult given the ongoing conflict, can you elaborate on how the ongoing conflict has affected you and your work?**	• Can you describe<br>o how you and your team feel working in [country]?<br>o any personal precautions you have taken as a result of the conflict?
	To learn about participant's work experience including	**1.3 Can you describe your experience working in**	• Can you describe what your position was in [country]? Can

	• their experience in conflict<br>• their experience in their current position	**conflict before entering [country]?**	you tell me how long you were stationed there?
	To learn about participant's educational background	**1.4 Can you describe your educational background?**	
2 *Objective: To understand what interventions are delivered by [UN agency]*	To identify what interventions were delivered	**2.3 We have been looking at [UN agency]'s activities in [country] and have made a list of the services or programs that [UN agency] delivers, could I share this list with you. [Share list of services]. Is there anything missing from the list or anything listed that is no longer delivered?**	
	To identify any gaps across the continuum of care	**2.4 So we have discussed [list services or interventions] as part of the services you provide. If you don't mind I'm going to list a few other areas of health. Could you tell me if your organization provides any services or interventions related to that area? [list continuum of care areas that weren't listed in 2.1 or found in document review, probe a few**	• Can you explain what considerations factored into the decision not to provide [ ] service?<br><br>Breastfeeding and IYCF probes, please inquire if there were any:<br><br>• interventions supporting milk expression?<br><br>• facilities where mothers can store milk?

		**examples if they say no interventions, however, do not probe on services that are outside of an organizations mandate] [ensure that you ask about breastfeeding promotion and IYCF]**	• interventions supporting non-breast fed children?<br>o E.g. Wet-nursing or informal milk sharing?<br>• interventions to support complementary feeding of children from 6 to 24 months?<br>• interventions to support micronutrient supplementation of children from 6 to 24 months?
3. *Objective: To understand how decisions are made to prioritize certain interventions*<br>*3.1 To understand how evidence was used to inform decision making*<br>*3.2 To understand how the availability of resources influenced prioritization*<br>*3.3 To understand how organization expertise influenced selection of interventions*	To learn, in the participant's own words, what they consider when prioritizing an intervention	**3.1. Just as a reminder, we're focusing on the period between [country specific time period]. Could you describe what informed and/or continues to inform your decisions on which interventions to deliver?**	
	To understand the role of surveys and data on service prioritization while paying particular attention to:<br>• health domain<br>• geographic variations<br>• populations (include sub-populations such as	**3.2. Between [country specific time period], were any formal or informal needs assessments or surveys conducted to determine health priorities?**<br>**3.3. Can you describe how surveys, surveillance data, results of these assessments, or other**	• What areas of health did these assessments focus on?<br>• What type of data was collected in these assessments?<br>• Can you describe the geographic areas where these assessments were conducted?

3.4 *The role of competing priorities*<br><br>Conceptual framework areas of investigation:<br>• Prioritization<br>• Financing<br>• Health workforce<br>• Commodities and Resources	ethnic or religious minorities)	**data influenced the interventions [UN agency] provides [in country]?**	• How was the decision made to conduct assessments in those specific geographic areas?<br>• Can you describe the population assessed (e.g. IDPs, refugees, non-displaced etc.)?<br>• How was the decision made to assess that specific population?<br>• Can you describe how often these assessments were conducted?<br>• Can you give me more information about who conducted these assessments?<br>• Would we be able to access these assessments?<br>• Can you describe any formal or informal evaluations of your programs influenced intervention priorities?
	To understand the role of the literature, guidelines, and cost-effectiveness on service prioritization	**3.4. Can you describe how scientific or academic literature influenced the interventions [UN agency] provides [in country]?**	• Can you list which guidelines you or your organizations uses? (If unfamiliar ask for access to these guidelines)

		**3.5. Can you describe how guidelines influenced the interventions [UN agency] provides [in country]?**<br><br>**3.6. Can you describe how the cost-effectiveness of different interventions influenced the interventions [UN agency] provides [in country]?**	
	To understand the role of the availability of health workers on health priorities while paying particular attention to:<br>• health domain<br>• geographic variations	**3.7. During [country specific time period], can you describe how the availability of certain cadres of health workers influenced intervention priorities?**	• Can you describe<br>o any issues you had with retaining your workforce? How did you manage these issues?<br><br>o the health worker recruitment and training process? Can you explain any issues you faced during recruitment?<br><br>o any interventions you had hoped to prioritize but couldn't because of a lack of available workforce?<br><br>o any interventions that weren't priorities but were

			delivered because of the available workforce?<br><br>• Did workforce availability affect<br>o one health domain in particular? Can you give me an example?<br>o one geographic area in particular? Can you give me an example?
	To understand how the availability of commodities influenced health priorities while paying particular attention to:<br>• health domain	**3.8 Between [country specific time period], were there any concerns about the availability or shortages of commodities? Can you give me an example?**<br><br>**3.9 Can you explain how the availability of certain commodities influenced intervention prioritization?**	• Can you describe<br>o any adjustments that had to be made to the interventions provided as a result of commodity availability? Can you give me an example?<br>o how stockpiles influenced intervention priorities?<br><br>• Did [UN agency] receive any commodity donations for use in [country]? Can you explain how these influenced intervention priorities?<br><br>• Did commodity availability affect one health domain in particular? Can you give me an example?

	To understand the role of funding and finances on health priorities while paying particular attention to:<br>• health domain	**3.10 Focusing on the period between [country specific time period], can you explain how the level of funding received by [UN agency] for work in [country] affected what health interventions were prioritized?**	• Can you describe<br>o the role that your ability to liquidate funds played in addressing the intervention priorities?<br>o the primary sources of funding?<br>• How did the sources of funding influence the types of services delivered? Were there any restrictions associated with this funding?<br>• Were there any situations when you had to contact head office about additional funds? Can you describe this situation for me?<br>• Were certain services deprioritized because of financial constraints? Can you give me an example?
	To understand how the availability of other elements or resources influenced health priorities while paying particular attention to:<br>• health domain	**3.11 Can you describe any other elements or resources that influenced the prioritization of interventions?**	• Did you have any difficulties procuring these resources? How did you address these difficulties?<br>• How did the shortages of [resource listed] influence the intervention? Were any adjustments made to the intervention due to [resource listed] shortages?

			• Were certain services deprioritized because of the availability of [resource mentioned]? Can you give me an example?
	To understand the role of organizational expertise on decision making	**3.12 Does [UN agency] have an expertise in one area of health?**<br><br>**3.13 How did [UN agency]'s expertise influence what interventions/health areas were identified as priorities?**	• Does your country team have expertise in one area of health? How has that influenced health intervention prioritization?
	To understand if and how increased attention to one area of health diverted attention from another health area	**3.14 Can you describe how you negotiate competing priorities? Can you give me an example?**	• In what sense are priorities competing? (i.e. time, workforce, resources, capital etc.)
4. *Objective: To understand how actors coordinate with one another*<br><br>Conceptual framework areas of investigation:<br>• Coordination<br>• Government legislation and restrictions	To understand the role of cluster meetings on intervention provision	**4.1 In your capacity as [position] with [UN agency] have you participated in any cluster meetings? If so within which clusters?**<br><br>**4.2 Could you explain what decisions were made at these meetings? Can you give me examples?**	• Could you describe the extent to which you interacted with the groups at the cluster meeting after the meetings?<br><br>• What are the differences between the services you provide and the services other members of the cluster group provide?<br><br>• How are/were these decisions made?

	To understand how organizations work with one another outside of the cluster system	**4.3 Outside of the NGOs that attend the cluster meetings does your organization work with any other organizations (local and international)?**	• Does [UN agency] subcontract services to other organizations?<br>o What types of services are subcontracted?<br>o Where are services subcontracted?<br>o Can you explain why services are subcontracted? Can you give me an example?<br>o Can you describe to what extent the subcontracted organization can influence the services or types of services delivered?
	To understand how the UN coordinates with government (including the level of government)	**4.4 Can you explain how your organization interacts with the government?**<br>**4.5 Whose responsibility is it to coordinate with government agencies? With whom do you interact within the government?**<br>**4.6 What level of government agencies do you coordinate with?**	• Can you describe how, if at all, [UN agency] coordinates their service delivery with government services?<br>• Could you describe any specific government requirements that [UN agency] must adhere to if they want to work within the country? Can you give me an example?<br>• Were there any activities that the government did not want you to

			report on? Could you give me an example? Can you explain this situation?<br><br>• Can you describe any laws or regulations that restricted your activities within the country? How did you manage these restrictions? (e.g. abortion restrictions)<br><br>• How did you manage constraints created by the government in a way that allowed [UN agency] to do their work?<br><br>• Does the UN allocate any funding to the government? Can you elaborate on the details of this funding?
5. Objective: To understand the contextual factors influencing the delivery of health interventions<br><br>Conceptual framework areas of investigation:<br>• Security context<br>• Sociocultural context	To unpack the impact of insecurity and conflict	**5.1 Can you briefly overview the current security situation in [country]? (e.g. access, incidents)**<br><br>**5.2 Can you describe the impact of security constraints on your work? Can you give me some examples?**<br><br>**5.3 Can you describe how your team managed security concerns?**	• Were visas or special permissions required for personnel entrance into the country or for the deployment of commodities, medications, or equipment?<br>o From whom were permissions sought?<br><br>• Were permissions required for the deployment of facilities or service providers?<br>o From whom were permissions sought?

			• To what extent were security considerations a basis for confining services to specific locations, such as IDP or refugee camp settings?<br><br>• Did the host government influence the location of health facilities or services?<br><br>• With whom did the providers of services negotiate security?<br><br>• Did security forces accompany the transport of personnel, commodities, or equipment to service locations?<br><br>• What groups provided security for health facilities or providers of services?<br><br>• Have security forces<br>o influenced where or what services were provided?<br>o influenced which populations or patients were to be provided services?

			o screened recipients of services before or at the service facility?<br>o interfered with the provision of services?<br>• Have any humanitarian workers or facilities been victims of violence or received threats of violence? If so, from whom (if known)<br>• Were non-standard fees or covert payments required to operate facilities?<br>• Was there any coordination with any opposition forces/tribal councils/other groups/country-specific category to allow the delivery of your intervention?

	To unpack the role of the sociocultural context on the delivery of interventions	**5.4 Can you explain how the sociocultural context (e.g. gender, caste, ethnicity etc.) influenced intervention selection/prioritization or delivery? Can you give me an example?**	• Were there any subpopulations that were particularly hard to access? Can you describe this group?<br><br>• Were there any special efforts to try to access this subpopulations? Can you give an example?<br><br>• Were different delivery methods used for different services in the same region? Can you explain what is underlying this difference?<br><br>• Did the sociocultural context affect one health domain in particular? Can you give me an example?<br><br>(context specific probes will need to be generated by each country team)
	To describe any other enabling and constraining factors not mentioned	**5.5 Can you describe any other factors that affected the delivery or implementation of interventions?**	
6. Objective: To understand changes in the set of interventions delivered<br><br>Conceptual framework areas of investigation:<br>• Finances<br>• Health workforce	To understand how the provided interventions have changed paying particular attention to the impact of:<br>• Funding changes<br>• Changes in security context	**6.1 Were there any changes to the services that were planned compared to those that were delivered?**	• How did fluctuations in the level of funding affect service provision?<br><br>• Can you describe the impact of the changing security context on the interventions delivered?

• Commodities and resources<br>• Security context		**6.2 Can you describe how these services changed?**<br><br>**6.3 What factors influenced changes?**	• Were any formal or informal evaluations conducted? Did these indicate the need for a change in the interventions provided? Could you give me an example?<br><br>• Were there any substantial influxes of displaced populations?<br><br>o Can you describe how this influx influenced intervention priorities? Can you give me an example?<br><br>o Did [UN agency] receive additional funds to provide services to the influx of population?<br><br>• Were there any epidemics while you were in the field?<br><br>o How did this shift priorities? Can you give me an example?<br><br>o Did [UN agency] receive additional funds to address the epidemic?<br><br>o Can you describe how long the effects of epidemic were felt?

			o Can you describe what happened with health services once the epidemic was addressed?<br><br>• Were there any natural disasters while you were in the field?<br><br>o How did this shift priorities? Can you give me an example?<br><br>o Did [UN agency] receive additional funds to address the impact of the natural disaster?<br><br>o Can you describe how long the effects of the natural disaster were felt?<br><br>o Can you describe what happened with health services once the situation with the natural disaster was resolved?

Interview Guide:
Governing Entity Official (i.e. Government or opposition)

I: Thank you for sitting down with me today: as you know, we are interested in understanding Sexual, Reproductive, Maternal, Newborn, Child, Adolescent Health & Nutrition intervention delivery in conflict. We know conflict is a particularly difficult context to work in, and our effort is focused on understanding how you deliver health services in these difficult contexts. We are speaking to a range of individuals involved in health service provision during conflict including different levels of NGO staff, UN officials, and government officials. In [country] we are focusing on the period between [country specific time period]. Before we begin I have a consent form that I would like you to sign. You can take as much time as you need to read over the form but I wanted to highlight a few items. First, ensuring your privacy is important to us, and we will remove all personal identifiers from our data. All recordings and notes will be assigned a code so that they are not linked to your name. The key to the codes will be kept in a password protected file which only the research staff can access. We will not link your name, or your position at your organization with your data in any of our documentation. Second, if at any time during the interview you feel uncomfortable and would like to halt the interview please let me know. I will give you a moment to look over the form, feel free to ask me any questions. When you feel comfortable doing so please sign the form. Let me know if you have any questions about the form.

give respondent time to sign the form, once form has been signed

I: This interview is being audio recorded. This is optional. If you do not want to be recorded, let me know and I will take detailed notes instead. This recording is for our records, and will only be shared with the research team. Do I have permission to record the interview?

if agrees put on recorder

if does not agree
I: That’s okay I can take written notes instead, do let me know if you change your mind at any point.

I: Do you have any questions before we begin?

General Objective	Detailed objectives	Interview Questions	Probes

3. *Objective: To establish rapport and learn about participant's work experience*	To understand participant's role in [Governing Entity] including<br>• Position within hierarchy (i.e. who they report to, and who reports to them)<br>• Responsibilities (e.g. allocation of finances, resources, etc.)<br>• Geographic areas they are responsible for	**1.1 Can you explain to me what your role as [position] with [Governing Entity] entails?**	• How do you interact with<br>o [Governing Entity]'s regional/provincial/district offices?<br>o [Governing Entity]'s head office?<br>• Can you describe your role<br>o in the allocation of finances?<br>o the allocation of resources and commodities?<br>o in determining what geographic areas [Governing Entity] works in?<br>• Can you explain what geographic areas you are responsible for in your position as [role]?
	To build rapport and understand the participant's general experience working in conflict in [country]	**1.2 We understand that working in [country] can be quite difficult given the ongoing conflict. Can you elaborate on how the ongoing conflict has affected you and your work?**	• Can you describe any personal precautions you have taken as a result of the conflict?
	To learn about participants' work experience	**1.3 Can you describe your work experience before**	

		**entering your role as [role]?**	
	To learn about participant's educational background	**1.4 Can you describe your educational background?**	
2 *Objective: To understand what interventions are delivered by [Governing Entity]*	To identify what interventions were delivered	**2.5 We have been looking at [Governing Entity]'s activities and have made a list of the services or programs that [Governing Entity] delivers. Could I share this list with you? [Share list of services]. Is there anything missing from the list or anything listed that is no longer delivered?**	
	To identify any gaps across the continuum of care	**2.6 So we have discussed [list services or interventions] as part of the services you provide. If you don't mind I'm going to list a few other areas of health. Could you tell me if the government provides any services or interventions related to these additional areas? [list continuum of care areas that weren't listed in 2.1 or found in document review, probe a few examples if they say no interventions, [ensure**	• Can you explain what considerations factored into the decision not to provide [ ] service?<br><br>Breastfeeding and IYCF probes, please inquire if there were any:<br><br>• interventions supporting milk expression?<br>• facilities where mothers can store milk?<br>• interventions supporting non-breast fed children?

		**that you ask about breastfeeding promotion and IYCF]**	o E.g. Wet-nursing or informal milk sharing?<br>• interventions to support complementary feeding of children from 6 to 24 months?<br><br>• interventions to support micronutrient supplementation of children from 6 to 24 months?
4. *Objective: To understand how decisions are made to prioritize certain interventions*<br>*4.1 To understand how evidence was used to inform decision making*<br>*4.2 To understand how the availability of resources influenced prioritization*<br>*4.3 The role of competing priorities*	To learn, in our participants' own words, what they consider when prioritizing an intervention	**3.1. Just as a reminder, we're focusing on the period between [country specific time period]. Could you describe what informed and/or continues to inform your decisions on which interventions to deliver?**	
	To understand the role of surveys and data on service prioritization while paying particular attention to:<br>• health domain<br>• geographic variations<br>• populations (include sub-populations such as	**3.8. Between [country specific time period], were any formal or informal needs assessments or surveys conducted to determine health priorities?**<br><br>**3.9. Can you describe how surveys, surveillance data, results of these assessments, or other**	• If no assessments were done, why? (e.g. resources, political concerns, etc.)<br><br>• What areas of health did these assessments focus on?<br><br>• What type of data was collected in these assessments?

Conceptual framework areas of investigation:<br>• Prioritization<br>• Financing<br>• Health workforce<br>• Commodities and Resources	ethnic or religious minorities)	**data influenced the interventions [Governing Entity] provides?**	• Can you describe the geographic areas where these assessments were conducted?<br><br>• How was the decision made to conduct assessments in those specific geographic areas?<br><br>• Can you describe the population assessed (e.g. IDPs, refugees, non-displaced etc.)?<br><br>• How was the decision made to assess that specific population?<br><br>• Can you describe how often these assessments were conducted?<br><br>• Can you share more information about who conducted these assessments?<br><br>• Would we be able to access these assessments?<br><br>• Can you describe how any formal or informal evaluations of your programs influenced intervention priorities?
	To understand the role of the literature, guidelines, and cost-effectiveness on service prioritization	**3.10. Can you describe how scientific or academic literature influenced the**	• Can you list which guidelines the government uses? (If unfamiliar ask for access to these guidelines)

		**interventions [Governing Entity] provides?**<br><br>**3.11. Can you describe how guidelines influenced the interventions [Governing Entity] provides?**<br><br>**3.12. Can you describe how the cost-effectiveness of different interventions influenced the interventions [Governing Entity] provides [in country]?**	
	To understand the role of the availability of health workers on health priorities while paying particular attention to:<br>• health domain<br>• geographic variations	**3.13. During [country specific time period], can you describe how the availability of certain cadres of health workers influenced intervention priorities?**	• Can you describe<br>o any issues you had with retaining the workforce? How did you manage these issues?<br><br>o the health worker recruitment and training process? Can you explain any issues you faced during recruitment?<br><br>o any interventions you had hoped to prioritize but couldn't because of a lack of available workforce?

			◦ any interventions that weren't priorities but were delivered because of the available workforce?<br><br>• Did workforce availability affect<br>◦ one health domain in particular? Can you give me an example?<br><br>◦ one geographic area in particular? Can you give me an example?
	To understand how the availability of commodities influenced health priorities while paying particular attention to:<br>• health domain	**3.8 Between [country specific time period], were there any concerns about the availability or shortages of commodities? Can you give me an example?**<br><br>**3.9 Can you explain how the availability of certain commodities influenced intervention prioritization?**	• Can you describe<br><br>◦ any adjustments that had to be made to the interventions provided as a result of commodity availability? Can you give me an example?<br><br>◦ how stockpiles influenced intervention priorities?<br><br>• Did [Governing Entity] receive any commodity donations for use in [country]? Can you explain how these influenced intervention priorities?<br><br>• Did commodity availability affect one health domain in particular? Can you give me an example?

	To understand the role of funding and finances on health priorities while paying particular attention to:<br>• health domain	**3.11 Focusing on the period between [country specific time period], can you explain how the level of funding received by [Governing Entity] affected what health interventions were prioritized?**	• Can you describe the primary sources of funding?<br><br>• How did the sources of funding influence the types of services delivered? Were there any restrictions associated with this funding?<br><br>• Were certain services deprioritized because of financial constraints? Can you give me an example?
	To understand how the availability of other elements or resources influenced health priorities while paying particular attention to:<br>• health domain	**3.15 Can you describe any other elements or resources that influenced the prioritization of interventions?**	• Did you have any difficulties procuring these resources? How did you address these difficulties?<br><br>• How did the shortages of [resource listed] influence the intervention? Were any adjustments made to the intervention due to [resource listed] shortages?<br><br>• Were certain services deprioritized because of the availability of [resource mentioned]? Can you give me an example?

	To understand if and how increased attention to one area of health diverted attention from another health area	**3.16 Can you describe how you negotiate competing priorities? Can you give me an example?**	• In what sense are priorities competing? (i.e. time, workforce, resources, capital etc.)
5. *Objective: To understand how actors coordinate with one another*<br><br>Conceptual framework areas of investigation:<br>• Coordination<br>• Government legislation and restrictions	To understand the role of cluster meetings on intervention provision	**4.1 In your capacity as [position] with [Governing Entity] have you participated in any cluster meetings? If so within which clusters?**<br><br>**4.2 Could you explain what decisions were made at these meetings? Can you give me examples?**	• Could you describe the extent to which you interacted with the groups at the cluster meeting after the meetings?<br><br>• What are the differences between the services you provide and the services other members of the cluster group provide?
	To understand how the government works with NGOs outside of the cluster system	**4.3 Outside of the NGOs that attend the cluster meetings, as [role] with [Governing Entity] do you work with any other organizations?**	• Does the government subcontract services to other organizations?<br><br>o What types of services are subcontracted?<br><br>o Where are services subcontracted?<br><br>o Can you explain why services are subcontracted? Can you give me an example?<br><br>o Can you describe to what extent the subcontracted organization can influence

			the services or types of services delivered?
	To understand how the government coordinates with UN	**4.7 Outside of your interactions during cluster meetings, can you list the UN agencies [Governing Entity] interacts with?**<br><br>**4.8 Can you explain to what extent [Governing Entity] interacts with [UN agency: ask for all UN agencies listed in response to 4.4]?**<br><br>**4.9 Within [Governing Entity] whose responsibility is it to coordinate with the UN agency [UN agency: ask for all UN agencies listed in response to 4.4]? With whom do you interact at [UN agency: ask for all UN agencies listed in response to 4.4]?**	• Could you describe any specific government requirements that [UN agency] must adhere to if they want to work within the country? Can you give me an example?
5. Objective: To understand the contextual factors influencing the delivery of health interventions<br><br>Conceptual framework areas of investigation:	To unpack the impact of insecurity and conflict	**5.5 Can you briefly overview the current security situation in [country]? (e.g. access, incidents)**<br><br>**5.6 Can you describe the impact of security constraints on your**	• Were visas or special permissions required for personnel entrance into the country or for the deployment of commodities, medications, or equipment?<br><br>o From whom were permissions sought?

• Security context<br>• Sociocultural context		**work? Can you give me some examples?**<br><br>**5.7 Can you describe how you manage security concerns?**	• Were permissions required for the deployment of facilities or service providers?<br>o From whom were permissions sought?<br><br>• To what extent were security considerations a basis for confining services to specific locations, such as IDP or refugee camp settings?<br><br>• What role did [Governing Entity] play in the location of health facilities or services?<br><br>• With whom did the providers of services negotiate security?<br><br>• Did security forces accompany the transport of personnel, commodities, or equipment to service locations?<br><br>• What groups provided security for health facilities or providers of services?<br><br>• Have security forces<br>o influenced where or what services were provided?

			- influenced which populations or patients were to be provided services? - screened recipients of services before or at the service facility? - interfered with the provision of services? - Have any humanitarian workers or facilities been victims of violence or received threats of violence? If so, from whom (if known) - Were non-standard fees or covert payments required to operate facilities? - Was there any coordination with any opposition forces/tribal councils/other groups/country-specific category to allow the delivery of your intervention?

	To unpack the role of the sociocultural context on the delivery of interventions	**5.8 Can you explain how the sociocultural context (e.g. gender, caste, ethnicity etc.) influenced intervention selection/prioritization or delivery? Can you give me an example?**	• Were there any subpopulations that were particularly hard to access? Can you describe this group?<br>• Were there any special efforts to try to access this subpopulations? Can you give an example?<br>• Were different delivery methods used for different services in the same region? Can you explain what is underlying this difference?<br>• Did the sociocultural context affect one health domain in particular? Can you give me an example?<br>• (context specific probes will need to be generated by each country team)
	To describe any other enabling and constraining factors not mentioned	**5.5 Can you describe any other factors that affected the delivery or implementation of interventions?**	
6. Objective: To understand changes in the set of interventions delivered<br><br>Conceptual framework areas of investigation:<br>• Finances<br>• Health workforce	To understand how the provided interventions have changed paying particular attention to the impact of:<br>• Funding changes<br>• Changes in security context	**6.1 Were there any changes to the services that were planned compared to those that were delivered?**<br><br>**6.2 Can you describe how these services changed?**	• How did fluctuations in the level of funding affect service provision?<br>• Can you describe the impact of the changing security context on the interventions delivered?

• Commodities and resources<br>• Security context		**6.3 What factors influenced changes?**	• Were any formal or informal evaluations conducted? Did these indicate the need for a change in the interventions provided? Could you give me an example?<br>• Were there any substantial influxes of displaced populations?<br>o Can you describe how this influx influenced intervention priorities? Can you give me an example?<br>o Did [Governing Entity] receive additional funds to provide services to the influx of population?<br>• Were there any epidemics while you were in the field?<br>o How did this shift priorities? Can you give me an example?<br>o Did [Governing Entity] receive additional funds to address the epidemic?<br>o Can you describe how long the effects of epidemic were felt?

			o Can you describe what happened with health services once the epidemic was addressed?<br><br>• Were there any natural disasters while you were in the field?<br>o How did this shift priorities? Can you give me an example?<br>o Did [Governing Entity] receive additional funds to address the impact of the natural disaster?<br>o Can you describe how long the effects of the natural disaster were felt?<br>o Can you describe what happened with health services once the situation with the natural disaster was resolved?

Interview Guide:
NGO (local and national) upper management

I: Thank you for sitting down with me today: as you know, we are interested in understanding Sexual, Reproductive, Maternal, Newborn, Child, Adolescent Health & Nutrition intervention delivery in conflict. We know conflict is a particularly difficult context to work in, and our effort is focused on understanding how you deliver health services in these difficult contexts. We are speaking to a range of individuals involved in health service provision during conflict including different levels of NGO staff, UN officials, and government officials. In [country] we are focusing on the period between [country specific time period]. Before we begin I have a consent form that I would like you to sign. You can take as much time as you need to read over the form but I wanted to highlight a few items. First, ensuring your privacy is important to us, and we will remove all personal identifiers from our data. All recordings and notes will be assigned a code so that they are not linked to your name. The key to the codes will be kept in a password protected file which only the research staff can access. We will not link your name, or your position at your organization with your data in any of our documentation. Second, if at any time during the interview you feel uncomfortable and would like to halt the interview please let me know. I will give you a moment to look over the form, feel free to ask me any questions. When you feel comfortable doing so please sign the form. Let me know if you have any questions about the form.

give respondent time to sign the form, once form has been signed

I: This interview is being audio recorded. This is optional. If you do not want to be recorded, let me know and I will take detailed notes instead. This recording is for our records, and will only be shared with the research team. Do I have permission to record the interview?

if agrees put on recorder

if does not agree

I: That’s okay I can take written notes instead, do let me know if you change your mind at any point.

I: Do you have any questions before we begin?

General Objective	Detailed objectives	Interview Questions	Probes
4. *Objective: To establish rapport and learn about participant's work experience*	To understand participant's role in [NGO] including<br>• Position within hierarchy (i.e. who they report to, and who reports to them)<br>• Responsibilities (e.g. allocation of finances, resources, etc.)<br>• Geographic areas they are responsible for	**4.1 Can you explain to me what your role as [position] with [NGO] entails?**	• How do you interact with<br>o [NGO]'s regional/provincial/district offices?<br>o [NGO]'s head office?<br>• Can you describe your role<br>o in the allocation of finances?<br>o the allocation of resources and commodities?<br>o in determining what geographic areas [NGO] works in?<br>• Can you explain what geographic areas you are responsible for in your position as [role]?
	To build rapport and understand the participant's general experience working in conflict in [country]	**4.2 We understand that working in [country] can be quite difficult given the ongoing conflict, can you elaborate on how the ongoing conflict has affected you and your work?**	• Can you describe<br>o how you and your team feel working in [country]?<br>o any personal precautions you have taken as a result of the conflict?
	To learn about participant's work experience including	**4.3 Can you describe your experience working in**	• Can you describe what your position was in [country]? Can

	• their experience in conflict<br>• their experience in their current position	**conflict before entering [country]?**	you tell me how long you were stationed there?
	To learn about participant's educational background	**1.4 Can you describe your educational background?**	
5. *Objective: To understand what interventions are delivered by [NGO]*	To identify what interventions were delivered	**2.7 We have been looking at [NGO]'s activities in [country] and have made a list of the services or programs that [NGO] delivers. Could I share this list with you? [Share list of services]. Is there anything missing from the list or anything listed that is no longer delivered?**	
	To identify any gaps across the continuum of care	**2.8 So we have discussed [list services or interventions] as part of the services you provide. If you don't mind I'm going to list a few other areas of health. Could you tell me if your organization provides any services or interventions related to that area? [list continuum of care areas that weren't listed in 2.1 or found in document review, probe a few examples. If they do not**	• Can you explain what considerations factored into the decision not to provide [ ] service?<br><br>Breastfeeding and IYCF probes, please inquire if there were any:<br><br>• interventions supporting milk expression?<br>• facilities where mothers can store milk?<br>• interventions supporting non-breast fed children?

		**report interventions, however, do not probe on services that are outside of an organization's mandate] [ensure that you ask about breastfeeding promotion and IYCF]**	o E.g. Wet-nursing or informal milk sharing?<br>• interventions to support complementary feeding of children from 6 to 24 months?<br>• interventions to support micronutrient supplementation of children from 6 to 24 months?
5. *Objective: To understand how decisions are made to prioritize certain interventions*<br>5.1 *To understand how evidence was used to inform decision making*<br>5.2 *To understand how the availability of resources influenced prioritization*<br>5.3 *To understand how organization expertise influenced selection of interventions*<br>5.4 *The role of competing priorities*	To learn, in our participant's own words, what they consider when prioritizing an intervention	**3.1. Just as a reminder, we're focusing on the period between [country specific time period]. Could you describe what informed and/or continues to inform your decisions on which interventions to deliver?**	
	To understand the role of surveys and data on service prioritization while paying particular attention to:<br>• health domain<br>• geographic variations<br>• populations (include sub-populations such as ethnic or religious minorities)	**3.14. Between [country specific time period], were any formal or informal needs assessments or surveys conducted to determine health priorities?**<br><br>**3.15. Can you describe how surveys, surveillance data, results of these assessments, or other data influenced the interventions [NGO] provides [in country]?**	• What areas of health did these assessments focus on?<br>• What type of data was collected in these assessments?<br>• Can you describe the geographic areas where these assessments were conducted?<br>• How was the decision made to conduct assessments in those specific geographic areas?

Conceptual framework areas of investigation:<br>• Prioritization<br>• Financing<br>• Health workforce<br>• Commodities and Resources			• Can you describe the population assessed (e.g. IDPs, refugees, non-displaced etc.)?<br><br>• How was the decision made to assess that specific population?<br><br>• Can you describe how often these assessments were conducted?<br><br>• Can you give me more information about who conducted these assessments?<br><br>• Would we be able to access these assessments?<br><br>• Can you describe any formal or informal evaluations of your programs influenced intervention priorities?
	To understand the role of the literature, guidelines, and cost-effectiveness on service prioritization	**3.16. Can you describe how scientific or academic literature influenced the interventions [NGO] provides [in country]?**<br><br>**3.17. Can you describe how guidelines influenced the interventions [NGO] provides [in country]?**	• Can you list which guidelines you or your organization uses? (If unfamiliar ask for access to these guidelines)

		**3.18. Can you describe how the cost-effectiveness of different interventions influenced the interventions [NGO] provides [in country]?**	
	To understand the role of the availability of health workers on health priorities while paying particular attention to:<br>• health domain<br>• geographic variations	**3.19. During [country specific time period], can you describe how the availability of certain cadres of health workers influenced intervention priorities?**	• Can you describe<br>o any issues you had with retaining the workforce? How did you manage these issues?<br>o the health worker recruitment and training process? Can you explain any issues you faced during recruitment?<br>o any interventions you had hoped to prioritize but couldn't because of a lack of available workforce?<br>o any interventions that weren't priorities but were delivered because of the available workforce?<br>• Did workforce availability affect<br>o one health domain in particular? Can you give me an example?

			o one geographic area in particular? Can you give me an example?
	To understand how the availability of commodities influenced health priorities while paying particular attention to:<br>• health domain	**3.8 Between [country specific time period], were there any concerns about the availability or shortages of commodities? Can you give me an example?**<br><br>**3.9 Can you explain how the availability of certain commodities influenced intervention prioritization?**	• Can you describe<br>o any adjustments that had to be made to the interventions provided as a result of commodity availability? Can you give me an example?<br>o how stockpiles influenced intervention priorities?<br>• Did [NGO] receive any commodity donations for use in [country]? Can you explain how these influenced intervention priorities?<br>• Did commodity availability affect one health domain in particular? Can you give me an example?
	To understand the role of funding and finances on health priorities while paying particular attention to:<br>• health domain	**3.12 Focusing on the period between [country specific time period], can you explain how the level of funding received by [NGO] for work in [country] affected what health**	• Can you describe<br>o the role that your ability to liquidate funds played in addressing the intervention priorities?<br>o the primary sources of funding?

		**interventions were prioritized?**	• How did the sources of funding influence the types of services delivered? Were there any restrictions associated with this funding?<br><br>• Were there any situations when you had to contact head office about additional funds? Can you describe this situation for me?<br><br>• Were certain services deprioritized because of financial constraints? Can you give me an example?
	To understand how the availability of other elements or resources influenced health priorities while paying particular attention to:<br>• health domain	**3.17 Can you describe any other elements or resources that influenced the prioritization of interventions?**	• Did you have any difficulties procuring these resources? How did you address these difficulties?<br><br>• How did the shortages of [resource listed] influence the intervention? Were any adjustments made to the intervention due to [resource listed] shortages?<br><br>• Were certain services deprioritized because of the availability of [resource mentioned]? Can you give me an example?

	To understand the role of organizational expertise on decision making	**3.18 Does [NGO] have an expertise in one area of health?**<br><br>**3.19 How did [NGO]'s expertise influence what interventions/health areas were identified as priorities?**	o Does your country team have expertise in one area of health? How has that influenced health intervention prioritization?
	To understand if and how increased attention to one area of health diverted attention from another health area	**3.20 Can you describe how you negotiate competing priorities? Can you give me an example?**	• In what sense are priorities competing? (i.e. time, workforce, resources, capital etc.)
6. *Objective: To understand how actors coordinate with one another*<br><br>Conceptual framework areas of investigation:<br>• Coordination<br>• Government legislation and restrictions	To understand the role of cluster meetings on intervention provision	**4.1 In your capacity as [position] with [their organization] have you participated in any cluster meetings? If so within which clusters?**<br><br>**4.2 Could you explain what decisions were made at these meetings? Can you give me examples?**	• Could you describe the extent to which you interacted with the groups at the cluster meeting after the meetings?<br><br>• What are the differences between the services you provide and the services other members of the cluster group provide?<br><br>• How were these decisions made?
	To understand how organizations work with one another outside of the cluster system	**4.3 Outside of the organizations that attend the cluster meetings does your organization work with any other organizations (local and international)?**	• Does your organization subcontract services to other organizations?<br><br>o What types of services are subcontracted?

			o Where are services subcontracted?<br><br>o Can you explain why services are subcontracted? Can you give me an example?<br><br>o Can you describe to what extent the subcontracted organization can influence the services or types of services delivered?<br><br>• Does your organization have services subcontracted to them by other organizations?<br><br>o What types of services are subcontracted to your organization?<br><br>o Where does your organization deliver these services?<br><br>o Can you describe to what extent your organization can influence the services or types of services delivered?
	To understand how NGOs coordinate with UN agencies	**4.10 Outside of your interactions during cluster meetings, can you list the**	

		**UN agencies your organization interacts with?**<br><br>**4.11 Can you explain to what extent your organization interacts with [UN agency: ask for all UN agencies listed in response to 4.4]?**<br><br>**4.12 Within your organization whose responsibility is it to coordinate with the UN agency [UN agency: ask for all UN agencies listed in response to 4.4]? With whom do you interact with at [UN agency: ask for all UN agencies listed in response to 4.4]?**	
	To understand how NGOs coordinate with government (including the level of government)	**4.13 Can you explain how your organization interacts with the government?**<br><br>**4.14 Whose responsibility is it to coordinate with government agencies? With whom do you**	• Can you describe how, if at all, your organization coordinates their service delivery with government services?<br><br>• Could you describe any specific government requirements that [NGO] must adhere to if they want to work within the country? Can you give me an example?

		**interact with in the government?**<br><br>**4.15 What level of government agencies do you coordinate with?**	• Were there any activities that the government did not want you to report on? Could you give me an example? Can you explain this situation?<br><br>• Can you describe any laws or regulations that restricted your activities within the country? How did you manage these restrictions? (e.g. abortion restrictions)<br><br>• How did you manage constraints created by the government in a way that allowed [NGO] to do their work?<br><br>• Does your organization receive any funding from the government? Can you elaborate on the details of this funding?
5. Objective: To understand the contextual factors influencing the delivery of health interventions<br><br>Conceptual framework areas of investigation:<br>• Security context<br>• Sociocultural context	To unpack the impact of insecurity and conflict	**5.9 Can you briefly overview the current security situation in [country]? (e.g. access, incidents)**<br><br>**5.10 Can you describe the impact of security constraints on your work? Can you give me some examples?**	• Were visas or special permissions required for personnel entrance into the country or for the deployment of commodities, medications, or equipment?<br>o From whom were permissions sought?

		**5.11 Can you describe how your team managed security concerns?**	• Were permissions required for the deployment of facilities or service providers?<br>o From whom were permissions sought?<br>• To what extent were security considerations a basis for confining services to specific locations, such as IDP or refugee camp settings?<br>• Did the host government influence the location of health facilities or services?<br>• With whom did the providers of services negotiate security?<br>• Did security forces accompany the transport of personnel, commodities, or equipment to service locations?<br>• What groups provided security for health facilities or providers of services?<br>• Have security forces<br>o influenced where or what services were provided?

			o influenced which populations or patients were to be provided services?<br>o screened recipients of services before or at the service facility?<br>o interfered with the provision of services?<br>• Have any humanitarian workers or facilities been victims of violence or received threats of violence? If so, from whom (if known)<br>• Were non-standard fees or covert payments required to operate facilities?<br>• Was there any coordination with any opposition forces/tribal councils/other groups/country-specific category to allow the delivery of your intervention?

	To unpack the role of the sociocultural context on the delivery of interventions	**5.12 Can you explain how the sociocultural context (e.g. gender, caste, ethnicity etc.) influenced intervention selection/prioritization or delivery? Can you give me an example?**	• Were there any subpopulations that were particularly hard to access? Can you describe this group?<br>• Were there any special efforts to try to access this subpopulations? Can you give an example?<br>• Were different delivery methods used for different services in the same region? Can you explain what is underlying this difference?<br>• Did the sociocultural context affect one health domain in particular? Can you give me an example?<br>(context specific probes will need to be generated by each country team)
	To describe any other enabling and constraining factors not mentioned	**5.5 Can you describe any other factors that affected the delivery or implementation of interventions?**	
6. Objective: To understand changes in the set of interventions delivered<br>Conceptual framework areas of investigation:<br>• Finances<br>• Health workforce	To understand how the provided interventions have changed paying particular attention to the impact of:<br>• Funding changes<br>• Changes in security context	**6.1 Were there any changes to the services that were planned compared to those that were delivered?**<br>**6.2 Can you describe how these services changed?**	• How did fluctuations in the level of funding affect service provision?<br>• Can you describe the impact of the changing security context on the interventions delivered?

• Commodities and resources<br>• Security context		**6.3 What factors influenced changes?**	• Were any formal or informal evaluations conducted? Did these indicate the need for a change in the interventions provided? Could you give me an example?<br><br>• Were there any substantial influxes of displaced populations?<br><br>o Can you describe how this influx influenced intervention priorities? Can you give me an example?<br><br>o Did [NGO] receive additional funds to provide services to the influx of population?<br><br>• Were there any epidemics while you were in the field?<br><br>o How did this shift priorities? Can you give me an example?<br><br>o Did [NGO] receive additional funds to address the epidemic?<br><br>o Can you describe how long the effects of epidemic were felt?

			o Can you describe what happened with health services once the epidemic was addressed?<br><br>• Were there any natural disasters while you were in the field?<br><br>o How did this shift priorities? Can you give me an example?<br><br>o Did [NGO] receive additional funds to address the impact of natural disaster?<br><br>o Can you describe how long the effects of the natural disaster were felt?<br><br>o Can you describe what happened with health services once the situation with the natural disaster was resolved?

BRANCH Country Case Study Common Protocol- Appendix 2: Intervention Coverage Indicators

Sexual and Reproductive Health	Name	Indicator	Numerator	Denominator	Source of Definition
1	Demand for family planning satisfied	Percentage of women aged 15-49, either married or in union, who have their need for family planning satisfied	Number of women aged 15-49, either married or in union, who are currently using any method of contraception	Total number of women who are married/in-union and who are currently using any method of contraception or who are fecund and not using any method of contraception but report wanting to space their next birth or stop childbearing altogether	Countdown to 2030
2	Contraceptive prevalence rate, modern methods	Percentage of women aged 15-49, either married or in union, who are using a modern contraceptive method at a particular point in time	Number of women aged 15-49, either married or in union, who are currently using a modern contraceptive method	Total number of women aged 15-49 who are married/in-union	MEASURE Evaluation
3	Method mix (i.e. contraceptive users, by method)	Percentage of women aged 15-49, either married or in union and are contraceptive users, who are using each modern method of contraception	Number of women aged 15-49, either married or in union and are contraceptive user by method	Total number of women aged 15-49, either married or in union and are contraceptive users	MEASURE Evaluation
4	Total fertility rate	Number of children who would be born per woman (or per 1,000 women) if she/they were to pass through the childbearing years bearing children according to a current schedule of age-specific fertility rates.	TFR = 5 ∑ ASFRa (for 5-year age groups) Where: ASFRa = age-specific fertility rate for women in age group a (expressed as a rate per woman).		MEASURE Evaluation
**Maternal Health and Pregnancy**	**Name**	**Indicator**	**Numerator**	**Denominator**	**Source of Definition**
5	Maternal mortality ratio	Number of maternal deaths per 100,000 live births	Number maternal deaths (i.e. while pregnant or within 42 days of the end of pregnancy, from any cause related to or aggravated by the pregnancy or its management but not from accidental or incidental causes) occurring within a reference period	Total number of live births	MEASURE Evaluation
6	Maternal case fatality ratio	Proportion of women with major obstetric complications who die in a facility within a reference period	Number of deaths from specified obstetric complications in a facility [Major complications include Hemorrhage: antepartum, intrapartum or postpartum; Prolonged/obstructed labor; Postpartum sepsis; Complications of abortion; Pre-eclampsia/eclampsia; Ectopic pregnancy; and Ruptured uterus.]	Total number of women with specified obstetric complications attended in the facility	MEASURE Evaluation
7	Stillbirth rate	The number of stillbirths per 1000 births (live and stillbirths)	Number of infants born per year with no sign of life and born after 28 weeks gestation, or weighing over 1000 g	Total number of births (live and stillbirths)	MEASURE Evaluation
8	Antenatal care (one or more visits)	Percentage of women attended to at least once during pregnancy by skilled health personnel for reasons related to the pregnancy	Number of women attended to at least once during pregnancy by skilled health personnel (doctor, nurse, midwife, or auxiliary midwife) for reasons related to the pregnancy	Total number of women who had a live birth	Countdown to 2030

9	Antenatal care (four or more visits)	Percentage of women attended to at least four or more times during pregnancy by any provider (skilled or unskilled) for reasons related to the pregnancy	Number of women attended to at least four times during pregnancy by any provider (skilled or unskilled) for reasons related to pregnancy	Total number of women who had a live birth	MEASURE Evaluation
10	Iron and Folate Supplementation	Percentage of pregnant women who received iron and folate supplementation	Number of pregnant women who received iron folate (60 mg iron + 400 µg folic acid) daily for 6 months	Total number of pregnant women	MEASURE Evaluation
11		Percentage of ANC clients who received iron and folate supplementation	Number of ANC clients who received iron folate (60 mg iron + 400 µg folic acid) daily for 6 months	Total number of ANC clients	MEASURE Evaluation
12	Skilled Birth Attendance	Percentage of women whose deliver was attended by a skilled health personnel	Number of women with a delivery (resulting in a live birth) attended by a skilled health personnel	Total number of women with a live birth	Countdown to 2030
13	Institutional Delivery	Percentage of births in all health facilities in the area	Number of deliveries in health facilities	Total number of women with a live birth	MEASURE Evaluation
14	Postnatal Care (mother)	Percentage of mothers who received postnatal care within two days of childbirth	Number of women who received a health check while in facility or at home following delivery, or a postnatal care visit within 2 days after delivery	Total number of women with a live birth (regardless of place of delivery)	Countdown to 2030
15	Caesarean Section Rate	Percentage of live births delivered by Caesarean section	Number of women with a live birth delivered by caesarean section	Total number of women with a live birth	Countdown to 2030
16	Intermittent preventive treatment for malaria during pregnancy	Percentage of women at-risk for malaria who received intermittent preventive treatment for malaria during pregnancy	Number of women at risk for malaria who received 4 or more doses of IPTp during pregnancy	Total number of women at risk for malaria with a live birth	World Health Organization. "WHO policy brief for the implementation of intermittent preventive treatment of malaria in pregnancy using sulfadoxine-pyrimethamine (IPTp-SP)." Geneva: World Health Organization (2013).
17			Number of women at risk for malaria who received their first dose of IPTp between weeks 13-18 during pregnancy	Total number of women at risk for malaria with a live birth	
18		Percentage of ANC clients who received 2 or more doses of a recommended antimalarial drug treatment to prevent malaria during pregnancy	Number of ANC clients who received 2 or more doses of a recommended antimalarial drug treatment to prevent malaria during pregnancy	Total number of ANC clients	MEASURE Evaluation
19	Prevention of mother-to-child transmission of HIV	Percentage of HIV-positive pregnant women who received antiretrovirals to reduce the risk of mother-to-child transmission	Number of HIV-infected pregnant women who received antiretrovirals in the last 12 months to reduce mother-to-child transmission	Estimated unrounded number of HIV-positive pregnant women	Countdown to 2030, MEASURE Evaluation
20	Screening and treatment of syphilis	Percentage of pregnant women screened for syphilis	Total number of women with a live birth who were screened for syphilis	Total number of women who had a live birth	
21		Percentage of ANC clients screened for syphilis	Number of ANC clients who were screened for syphilis	Total number of ANC clients	MEASURE Evaluation
22	Active management of third stage of labour (to deliver the placenta) to prevent	Percentage of pregnant women who receive an uterotonic (oxytocin or misoprostol) in the third stage of labor or immediately after birth	Number of women who received an uterotonic (oxytocin or misoprostol) in the third stage of labor or immediately after birth.	Total number of women who gave birth at health facilities	Maternal Health Task Force

	postpartum hemorrhage (as above plus manual removal of placenta)				
23	Safe Abortion	Percentage of induced abortions managed using MVA or medical evacuation	Number of induced abortions managed using MVA or medical evacuation	Total pregnancy terminations (Indirect estimates)	
24	Neonatal tetanus protection	Percentage of newborns protected against tetanus	Number of women with a live birth who received two doses of tetanus toxoid vaccine within the appropriate interval prior to the infant's birth	Total number of women with a live birth	MEASURE Evaluation
25		Percentage of newborns, born to ANC clients, protected against tetanus	Number of ANC clients with a live birth who received two doses of tetanus toxoid vaccine within the appropriate interval prior to the infant's birth	Total number of ANC clients	
26	Detect and manage postpartum sepsis	Percentage of newly delivered mothers presenting with potential and potential organ dysfunction treated with IV fluids and antibiotic	Number of newly delivered mothers presenting with potential infection [fever or hypothermia] and potential organ dysfunction [fast heart rate, or low blood pressure, or respiratory distress, or jaundice, or decreased urination, or altered mental status] treated with IV fluids and antibiotic	Number of newly delivered mothers presenting with potential infection [fever or hypothermia] and potential organ dysfunction [fast heart rate, or low blood pressure, or respiratory distress, or jaundice, or decreased urination, or altered mental status]	Singer, Mervyn, et al. "The third international consensus definitions for sepsis and septic shock (sepsis-3)." Jama 315.8 (2016): 801-810.
27	Basic Emergency Obstetric and Newborn Care (BEmONC) availability	Percentage of health facilities providing BEmONC at least once in the previous three months per 500,000 population.	Number of facilities providing BEmONC [health facilities operating 24/7 and reporting provision of all 7 BEmONC signal functions [1) administer parenteral antibiotics 2) administer utertonic drugs (i.e. parenteral oxytocin) 3) administer parenteral anticonvulsants for preeclampsia and eclampsia (i.e. magnesium sulfate) 4) manually remove the placenta 5) remove retained products (e.g. vacuum extraction, dilation and curettage) 6) perform assisted vaginal delivery (e.g. vacuum extraction, forceps delivery) 7) perform basic neonatal resuscitation (e.g. with bag and mask)]]	The total population for the same administrative or health area, at the same point in time	Humanitarian Indicator Registry, MEASURE evaluation
28	Comprehensive Emergency Obstetric and Newborn Care (CEmONC) availability	Percentage of health facilities providing CEmONC at least once in the previous three months per 500,000 population.	Number of facilities providing CEmONC [health facilities operating 24/7 and reporting provision of BEmONC + surgical care and blood transfusion]	The total population for the same administrative or health area, at the same point in time	Humanitarian Indicator Registry, MEASURE evaluation
29	Low body mass index	Percentage of women aged 15-49 with a BMI of less than 18.5 kg/m^2	Number of women aged 15-49 with a BMI of less than 18.5 kg/m2 (excluding pregnant women)	Total number of women aged 15-49 (excluding pregnant women)	MEASURE Evaluation
**Newborn Health**	**Name**	**Indicator**	**Numerator**	**Denominator**	**Source of Definition**
30	Neonatal mortality rate	Number of neonatal deaths per 1000 live births	Number of neonatal deaths (0-27 days)	Total number of live births	
31	Early neonatal mortality rate	Number of early neonatal deaths per 1000 live births	Number of neonatal deaths (0-7 days)	Total number of live births	

32	Infant mortality rate (IMR)	Number of infant deaths per 1000 live births	Number of infants who died before their first birthday (0−11 months of age)	Total number of live births	
33	Postnatal Care (newborn)	Percentage of babies who received postnatal care within two days of childbirth	Number of live births who received a health check while in facility or at home following delivery within 2 days after delivery	Total number of live births	MEASURE Evaluation
34	Newborn resuscitation	Percentage of newborns who were resuscitated	Number of newborns for whom resuscitation (stimulation and/or bag and mask) was initiated	Total number of live births	Every Newborn Action Plan (ENAP)
35		Percentage of facilities that demonstrate neonatal resuscitation readiness (facilities with functional bag and mask for neonatal resuscitation in delivery room)	Number of facilities with a functional neonatal bag and two masks (sizes 0 and 1) in the labour and delivery service area	Total number of facilities with inpatient maternity services assessed	ENAP
36	Promotion and provision of thermal care for all newborns to prevent hypothermia (immediate drying, skin to skin, delayed bathing)	Percentage of births delivered at home or in facilities where the newborns were dried with cloth immediately after birth. (The term 'immediately' needs to be defined as a specific amount of time in minutes)	Number of newborns dried with cloth immediately after birth	Total number of live births	MEASURE Evaluation
37		Percentage of births delivered at home or in facilities, where the newborns had their first bath delayed at least six hours after birth. Ideally the bath should be delayed until 24 hours after birth	Number of newborns with first bath delayed at least six hours after birth	Total number of live births	MEASURE Evaluation
38	Promotion and provision of hygienic cord and skin care	Percentage of births delivered at home or in facilities where the newborn had nothing harmful applied to the umbilical cord after cutting and tying.	Number of newborns with nothing harmful applied to cord	Total number of live births	MEASURE Evaluation
39		Percentage of newborns who received chlorhexidine digluconate for umbilical cord care	Number of newborns who received chlorhexidine digluconate for umbilical cord care at birth within 24 hours of birth	Total number of live births	ENAP
40		Coverage of clean cord care at the time of delivery, either from use of a clean delivery kit, a new blade or instrument, or a boiled blade or instrument, to cut the baby's umbilical cord (home deliveries only)	Number of births with a clean delivery kit or clean blade (home deliveries only)	Total number of births (home deliveries only)	MEASURE Evaluation
41	Case management of neonatal sepsis, meningitis and pneumonia	Percentage of newborns treated for severe neonatal infection	Number of newborns who received at least one injection of antibiotic for possible serious bacterial infection in the facility	Total number of live births	ENAP
42		Percentage of facilities with treatment available for severe neonatal infection	Number of facilities in which gentamicin is available at suitable peripheral level for treatment of severe neonatal infection	Total number of facilities assessed	ENAP
43	Kangaroo Mother Care	Percentage of facilities demonstrating KMC readiness	Number of facilities in which a space is identified for KMC and where staff have received KMC training (< 2 years)	Total number of facilities assessed with inpatient maternity services	ENAP

44		Percentage of low birth weight infants with access to kangaroo mother care	Number of facility born low birth weight infants with access to kangaroo mother care	Total number of facility born low birth weight infants	ENAP
45	Early initiation of breastfeeding	Percentage of newborns put to the breast within one hour of birth	Number of women with a live birth who put the newborn infant to the breast within 1 hour of birth	Total number of women with a live birth	Countdown to 2030
46		Percentage of newborns put to the breast within one hour of birth	Number of women with a live birth who put the newborn infant to the breast within 1 hour of birth	Total number of women with a live birth in a facility	
**Child Health**	**Name**	**Indicator**	**Numerator**	**Denominator**	**Source of Definition**
47	U5MR (Under 5 mortality rate)	Number of child deaths per 1,000 live births	Number of deaths among children aged 0–59 months	Total number of live births	
48	Measles vaccination coverage	Percentage of children (12-23 months) who have received measles vaccination	Number of children (12-23 months) immunized with measles containing vaccine	Total number of children aged 12–23 months	
49		Percentage of children (6-59 months) who have received measles vaccination	Number of children (6-59 months) who have received measles vaccination in a given year	Total number of children aged 6-59 months	
50		Percentage of children (6m-15 yrs) who have received measles vaccination	Number of children (6m-15 yrs) who have received measles vaccination in a given year	Total number of children aged 6m-15 yrs	
51	Polio vaccination coverage	Percentage of children (12-23 months) who have received polio vaccination	Number of children (12-23 months) who survive the first year of life who have received at least three doses of polio vaccine	Total number of children aged 12–23 months	
52	Diphtheria with tetanus toxoid and pertussis vaccination coverage	Percentage of children (12-23 months) who have received combined diphtheria/pertussis/tetanus (DTP3) vaccination	Number of children (12-23 months) who received three doses of diphtheria/pertussis/tetanus vaccine	Total number of children aged 12–23 months	
53	Haemophilus influenza type B vaccination coverage	Percentage of children (12-23 months) who have received Haemophilus influenza type B immunization coverage (HiB3)	Number of children (12-23 months) who received three doses of Haemophilus influenza type B vaccine	Total number of children aged 12–23 months	
54	Pneumococcal conjugate vaccination coverage	Percentage of children (12-23 months) who have received pneumococcal conjugate vaccine	Number of children (12-23 months) who received the third dose of pneumococcal conjugate vaccine	Total number of children aged 12–23 months	
55	Fully Immunized	Percentage of children (12-23 months) who have received all recommended vaccinations (DTP3, polio, and measles)	Number of children (12-23 months) who have received all recommended vaccinations	Total number of children aged 12–23 months	
56	Care seeking for malaria	Percentage of children under five years old with fever in the last two weeks for whom advice or treatment was sought	Number of children under five years old who had a fever in the previous two weeks for whom advice or treatment was sought	Total number of children under five years old who had a fever in the previous two weeks	Household Survey Indicators for Malaria Control (MEASURE Evaluation, MEASURE DHS, President's Malaria Initiative, Roll Back Malaria Partnership UNICEF, World Health Organization)

57	Insecticide-treated net use	Percentage of children under 5 years sleeping under an insecticide-treated mosquito net	Number of children ages 0–59 months sleeping under an insecticide-treated mosquito net the night before	Total number of children ages 0–59	Countdown to 2030
58	Case management of childhood pneumonia	Percentage of children under 5 years with suspected pneumonia taken to an appropriate health provider	Number of children ages 0–59 months with suspected pneumonia in the previous two weeks who were taken to an appropriate health provider	Total number of children ages 0–59 months with suspected pneumonia in the previous two weeks	Countdown to 2030
59		Percentage of children under 5 years with suspected pneumonia receiving antibiotics	Number of children ages 0–59 months with suspected pneumonia in the previous two weeks receiving antibiotics	Total number of children ages 0–59 months with suspected pneumonia in the previous two weeks	Countdown to 2030
60	Case management of diarrhea a) acute watery diarrhea b) dysentery	Percentage of children under 5 years with diarrhea receiving oral rehydration therapy and continued feeding	Number of children ages 0–59 months with diarrhea in the previous two weeks receiving oral rehydration therapy (oral rehydration solution and/or recommended homemade fluids or increased fluids) and continued feeding	Total number of children ages 0–59 months with diarrhea the previous two weeks	Countdown to 2030
61		Percentage of children under 5 years with diarrhea receiving oral rehydration solution	Number of children ages 0–59 months with diarrhea in the previous two weeks receiving oral rehydration solution	Total number of children ages 0–59 months with diarrhea in the previous two weeks	MEASURE Evaluation
**Adolescent Health**	**Name**	**Indicator**	**Numerator**	**Denominator**	**Source of Definition**
62	Adolescent mortality rate	Number of deaths among adolescents (10-19 years) per 100 000 adolescent population	Number of deaths among adolescents aged 10-19	Number of adolescents aged 10-19	
63	Adolescent pregnancy rates	Percentage of adolescent females (10-19 years) who have ever been pregnant	Number of adolescent females (10-19 years) who report having ever been pregnant	Total number of adolescent females (10-19 years)	MEASURE Evaluation
64		Percentage of adolescent males (10-19 years) who have ever caused a pregnancy	Number of adolescent males (10-19 years) who report having ever caused a pregnancy	Total number of adolescent males (10-19 years)	MEASURE Evaluation
65	Adolescent birth rate	Number of births to women ages 15–19 per 1,000 women	Number of live births to females aged 15-19	Total number of adolescent females (15-19 years)	MEASURE Evaluation
66	Early marriage	Percentage of women age 20-24 who were married or in union before age 15	Number of women age 20-24 who were married or in union before age 15	Total number of women aged 20-24	UNICEF
67		Percentage of women 20-24 years old who were married or in union before age 18	Number of women 20-24 years old who were married or in union before age 18	Total number of women aged 20-24	UNICEF
**Nutrition**	**Name**	**Indicator**	**Numerator**	**Denominator**	**Source of Definition**
68	Exclusive breastfeeding (<6 months)	Percentage of infants (0–5 months) who are exclusively breastfed	Number of infants ages 0–5 months who are exclusively breastfed during the previous day	Total number of infants ages 0–5 months	Countdown to 2030
69	Introduction of solid, semi-solid/soft foods (6-8 months)	Percentage of infants (6–8 months) who receive solid, semi-solid or soft foods	Number of infants ages 6–8 months who received solid, semi-solid or soft foods during the previous day	Total number of infants ages 6–8 months	Countdown to 2030
70	Minimum acceptable diet	Percentage of children (6-23 months) who receive a minimum acceptable diet	Number of breastfed children 6-23 months of age who had at least the minimum dietary diversity and the minimum meal frequency during the previous day [Minimum dietary diversity: Child 6-	Total number of breastfed children 6-23 months of age	Humanitarian Indicator Registry

			23 months consumed foods from 4 or more food groups (out of 7 groups) during the previous day.]		
71			Number of non-breastfed children 6-23 months of age who received at least 2 milk feedings and had at least the minimum dietary diversity not including milk feeds and the minimum meal frequency during the previous day [Minimum meal frequency: Child receives solid, semi-solid, or soft foods (but also includes milk feed for non-breastfed children) the minimum number of times or more over the previous day. The minimum number of times are:<br>2 times for breastfed infants 6-8 months<br>3 times for breastfed children 9-23 months<br>4 times for non-breastfed children 6-23 months]	Total number of non-breastfed children 6-23 months of age	Humanitarian Indicator Registry
72	Vitamin A Supplementation	Percentage of children (6–59 months) who received two doses of vitamin A	Number of children aged 6–59 months who received two doses of vitamin A during the previous note	Total number of children ages 6–59 months	Countdown to 2030
73	SAM treatment	Proportion of cases (6-59 months) with severe acute malnutrition receiving treatment	Number of cases (6-59 months) with severe acute malnutrition receiving for treatment	Total number of cases (6-59 months) with severe acute malnutrition	Humanitarian Indicator Registry
74		Proportion of discharged cases (6-59 months) with severe acute malnutrition who recovered	Number of discharged cases (6-59 months) with severe acute malnutrition who recovered	Total number of cases (6-59 months) with severe acute malnutrition discharged	Humanitarian Indicator Registry
75		Proportion of discharged cases (6-59 months) with severe acute malnutrition who defaulted	Number of discharged cases (6-59 months) with severe acute malnutrition who defaulted	Total number of cases (6-59 months) with severe acute malnutrition discharged	Humanitarian Indicator Registry
76		Proportion of cases (6-59 months) with severe acute malnutrition who died during treatment	Number of cases (6-59 months) with severe acute malnutrition who died during treatment	Total number of cases (6-59 months) with severe acute malnutrition discharged	Humanitarian Indicator Registry
77		Proportion of discharged cases (6-59 months) with severe acute malnutrition who non-recovered	Number of discharged cases (6-59 months) with severe acute malnutrition who non-recovered	Total number of cases (6-59 months) with severe acute malnutrition discharged	Humanitarian Indicator Registry
78		Number of cases (6-59 months) with severe acute malnutrition referred to inpatient care or hospital	Number of cases (6-59 months) with severe acute malnutrition referred to inpatient care or hospital		Humanitarian Indicator Registry
79	GAM (disaggregate <6 months, 6-59 months)	Percentage of children with either MAM or SAM	Number of children aged 6-59 months with MAM or SAM	Total number of children aged 6–59 months	Humanitarian Indicator Registry
80	SAM (disaggregate <6 months, 6-59 months)	Percentage of children who are severely wasted children (<-3 Z-score)	Number of children aged 6-59 months who are severely wasted children with <-3 Z-score	Total number of children aged 6–59 months	Humanitarian Indicator Registry
81	MAM (disaggregate <6 months, 6-59 months)	Percentage of children who are moderately wasted children (-3 to -2 Z-score)	Number of children aged 6-59 months who are moderately wasted children with -3 to -2 Z-score	Total number of children aged 6–59 months	Humanitarian Indicator Registry
82	Stunting prevalence	Percentage of children falling into Z-score categories for height for age: <-3Z (severe stunting) and -3 to -2Z (moderate stunting)	Number of children aged 0–59 months who are stunted with <-2 Z-score	Total number of children aged 6–59 months	Humanitarian Indicator Registry

83	Anemia prevalence in women aged 15-49 (disaggregated by pregnancy status)	Percentage of non-pregnant women aged 15-49 with hemoglobin levels < 120 g/L.	Number of non-pregnant women aged 15-49 with hemoglobin levels < 120 g/L.	Total number of non-pregnant women aged 15-49 screened for hemoglobin levels	MEASURE Evaluation
84		Percentage of pregnant women with hemoglobin levels < 110 g/L.	Number of pregnant women with hemoglobin levels < 110 g/L.	Total number of pregnant women screened for hemoglobin levels	MEASURE Evaluation
85	Acute malnutrition in pregnant and lactating women (based on mid-upper arm circumference, MUAC)	Percentage of pregnant and lactating women with a MUAC under cut-off of <23 cm	Number of pregnant and lactating women with infants below age of 0 to 5 months that meet the criteria for acute malnutrition based on MUAC under cut-off of <23 cm	Total number of pregnant and lactating women with infants below age of 0 to 5 months	Humanitarian Indicator Registry
**Mental Health**	**Name**	**Indicator**	**Numerator**	**Denominator**	**Source of Definition**
86	Mental health specialist referral	Percentage of cases identified in need of psychosocial/mental health services who are referred to specialist services	Number of children identified as in need of specific psychosocial and mental health services who are referred to focused specialized services	Total number of children identified as in need of specific psychosocial and mental health services	Humanitarian Indicator Registry
**Gender based violence**	**Name**	**Indicator**	**Numerator**	**Denominator**	**Source of Definition**
87	Sexual Violence Incidence	Number of reported incidents of sexual violence per 10,000 population	Number of reported incidents of sexual violence in the area of analysis	Total population	Humanitarian Indicator Registry
88	Clinical management of sexual assault service availability	Percentage of functional health facilities providing services for clinical management of sexual assault	Number of functional health facilities, i.e. all public and private health facilities, with clinical management of sexual assault	Total number of functional health facilities	Humanitarian Indicator Registry
89	Clinical management of sexual assault health worker availability	Percentage of health workers trained on clinical management of rape survivors	Number of health staff trained on clinical management of rape	Total number of health workers (in the categories selected at the national level, e.g. medical officers, nurses, etc.)	Humanitarian Indicator Registry
90	Community-based psychosocial support for GBV health worker availability	Percentage of community-based workers trained in psychosocial support and referral pathways for GBV survivors	Number of community-based workers trained in GBV psychosocial support	Total number of community-based workers	Humanitarian Indicator Registry
91	Post-exposure prophylaxis (PEP) for rape survivors	Percentage of survivors who receive post exposure prophylaxis for the prevention of transmission of HIV	Number of survivors of rape who receive PEP within 72 hours of incident	Total number of reported incidents of rape	Humanitarian Indicator Registry
92	Intimate Partner Violence Prevalence	Percentage of ever-partnered women in a given population who have ever experienced physical and/or sexual violence by an intimate partner	Number of ever-partnered women in a given population who have ever experienced physical and/or sexual violence by an intimate partner	Total number of ever-partnered women in a given population	UN-Violence Against Women Expert Group
**WASH**	**Name**	**Indicator**	**Numerator**	**Denominator**	**Source of Definition**
93	Use of improved drinking water sources	Percentage of the population using improved drinking water sources	Number of household members using improved drinking water sources (including piped on premises, public standpipe, borehole, protected dug well, protected spring, rainwater collection)	Total number of household members	Countdown to 2030
94	Use of improved sanitation facilities	Percentage of the population using improved sanitation facilities	Number of household members using improved sanitation facilities (including connection to a public sewer, connection to a septic system, pour-flush latrine, pit latrine with a slab, or a ventilated	Total number of household members	Countdown to 2030

			improved pit latrine) not shared with other households		
**Injury**	**Name**	**Indicator**	**Numerator**	**Denominator**	**Source of Definition**
95	Access to timely essential surgery	Proportion of the population that can access, within 2 hours, a facility that can do caesarean delivery, laparotomy, and treatment of open fracture	Number of individuals who can access, within 2 hours, a facility that can do caesarean delivery, laparotomy, and treatment of open fracture	Total population	Lancet Commission on Surgery
96	Specialist surgical workforce density	Number of specialist surgical, anesthetic, and obstetric physicians who are working, per 100 000 population	The number of specialist surgical, anesthetic, and obstetric physicians	Total population	Lancet Commission on Surgery
97	Assistance to mine/ERW victims	Percentage of survivors from mine/ERW incidents receiving support	Number of survivors from mine/ERW incidents receiving emergency medical care and psychological support	Total number of survivors from mine/ ERW incidents	Humanitarian Indicator Registry
**Other**	**Name**	Indicator	Numerator	Denominator	
98	Cause specific mortality rates as available				
